# Proportions and incidence of locally advanced cervical cancer: a global systematic literature review

**DOI:** 10.1136/ijgc-2022-003801

**Published:** 2022-10-14

**Authors:** Bradley J Monk, David S P Tan, José David Hernández Chagüi, Jitender Takyar, Michael J Paskow, Ana Tablante Nunes, Eric Pujade-Lauraine

**Affiliations:** 1 Virginia G Piper Cancer Center at HonorHealth, Phoenix, Arizona, USA; 2 Division of Gynecologic Oncology, University of Arizona College of Medicine, Creighton University School of Medicine, Phoenix, Arizona, USA; 3 Department of Haematology-Oncology, National University Cancer Institute, Singapore; 4 Department of Medicine, Yong Loo Lin School of Medicine, Cancer Science Institute of Singapore, National University of Singapore, Singapore; 5 Global Medical Affairs, AstraZeneca, Gaithersburg, Maryland, USA; 6 Evidence Evaluation HEOR, Parexel International, Chandigarh, India; 7 Late Development Oncology, AstraZeneca, Gaithersburg, Maryland, USA; 8 ARCAGY-GINECO, Paris, France

**Keywords:** Cervix Uteri

## Abstract

**Background:**

Optimal treatment of cervical cancer is based on disease stage; therefore, an understanding of the global epidemiology of specific stages of locally advanced disease is needed.

**Objective:**

This systematic literature review was conducted to understand the global and region-specific proportions of patients with cervical cancer with locally advanced disease and to determine the incidence of the locally advanced disease.

**Methods:**

Systematic searches identified observational studies published in English between 2010 and June 10, 2020, reporting the proportion of patients with, and/or incidence of, locally advanced stages of cervical cancer (considered International Federation of Gynecology and Obstetrics (FIGO) IB2–IVA). Any staging criteria were considered as long as the proportion with locally advanced disease was distinguishable. For each study, the proportion of locally advanced disease among the cervical cancer population was estimated.

**Results:**

The 40 included studies represented 28 countries in North or South America, Asia, Europe, and Africa. Thirty-eight studies reported the proportion of locally advanced disease among populations with cervical cancer. The estimated median proportion of locally advanced disease among all cervical cancer was 37.0% (range 5.6–97.5%; IQR 25.8–52.1%); estimates were generally lowest in North America and highest in Asia. Estimated proportions of ≥50% were reported in nine studies from Asia, Europe, Brazil, and Morocco; estimates ≤25% were reported in six studies from Asia, United States, Brazil, and South Africa. Locally advanced disease was reported for 44% and 49% of women aged >70 and ≥60 years, and 5–100% of younger women with cervical cancer. A greater proportion of locally advanced disease was reported for Asian American (19%) versus White women (8%) in one United States study. Two of five studies describing the incidence of locally advanced disease reported rates of 2–4/100 000 women among different time frames.

**Conclusion:**

This review highlights global differences in proportions of locally advanced cervical cancer, including regional variance and disparities according to patient race and age.

**Highlights**

- The median proportion of locally advanced cervical cancer was 37.0% (IQR 26–52%).
- Proportions of locally advanced disease were highest in Asian countries and lowest in North American countries.
- Two studies reported incidence rates of 2–4/100 000 women among different time frames.

**WHAT IS ALREADY KNOWN ON THIS TOPIC**

- Cervical cancer is the fourth most common cancer among women; prevalence and incidence vary among countries and world regions. Epidemiologic studies do not frequently report cervical cancer by disease stage.

**WHAT THIS STUDY ADDS**

- In this systematic literature review, the median proportion of locally advanced cervical cancer was 37% (range 6–98%) across all studies. Lower proportions of locally advanced disease were observed in North America versus Asia, Europe, and other regions; Asia had the highest overall proportion.

**HOW THIS STUDY MIGHT AFFECT RESEARCH, PRACTICE OR POLICY**

- This study encourages policy change that supports early, consistent screening of at-risk populations.

## INTRODUCTION

Cervical cancer is the fourth most common cancer in women, and the first among gynecological cancers.^1^ Patients with locally advanced disease have 5- year disease-free survival of 68% and 5-year overall survival of 74%; advanced stage and lymph node involvement contribute to worse prognosis.^2^ Standard-of-care drug treatment has not changed for over 20 years, and the recent OUTBACK trial, which investigated the addition of adjuvant chemotherapy to standard-of-care chemoradiotherapy for locally advanced cervical cancer, did not improve outcomes compared with chemoradiation alone (5-year overall survival: 72% vs 71%; 5-year progression-free survival: 63% vs 61%).^3^ Because optimal treatment for cervical cancer is largely determined by disease stage, it is important to understand the proportions and incidence of specific stages of cervical cancer, and a global comparison is lacking. We conducted a systematic literature review of observational studies reporting the proportions and incidence of cervical cancer by disease stage to elucidate global and region-specific burden of locally advanced disease.

## METHODS

### Systematic Literature Searches and Data Extraction

This review was designed and conducted in accordance with the 2020 guidelines for Preferred Reporting Items for Systematic Reviews and Meta-Analyses (PRISMA). A completed PRISMA checklist is provided in the Online Supplemental Appendix. Systematic searches were conducted to identify observational studies published in English between 2010 and June 10, 2020, reporting proportions and/or incidence of locally advanced stages of cervical cancer. Locally advanced cervical cancer was defined as stages IB2–IVA according to any version of the International Federation of Gynecology and Obstetrics (FIGO) staging criteria; publications reporting stage by other criteria were included if the FIGO equivalent could be ascertained.

10.1136/ijgc-2022-003801.supp1 Supplementary data

Two reviewers screened all citations and full-text articles independently; any discrepancies were resolved by a third independent reviewer. Data from the included articles were independently extracted by two reviewers into a spreadsheet with predefined categories of information, and the results were checked and reconciled by a third independent reviewer. The proportion of patients with, and incidence of, locally advanced cervical cancer for the full study population as well as any subgroup analyses were extracted.

Additional eligibility criteria, search strategies, STROBE (Strengthening the Reporting of Observational studies in Epidemiology) checklists, and further details are provided in the Online Supplemental Methods and Online Supplemental Tables 1–4. Institutional review board approval was not obtained, as all data were extracted from previously published studies.

### Calculation of the Proportion of Locally Advanced Cervical Cancer

The percentages of patients with cervical cancer at different stages of disease were extracted from the included studies. Within each publication, disease stage was reported as (a) basic number without further detail (ie, I, II, III, IV); (b) basic category (eg, in situ, localized, regional, distant); or (c) full FIGO classification (eg, IA1, IIB, IVA). We used a conservative approach to calculate the proportions of locally advanced cervical cancer by only counting stages where a majority of patients would be considered to have locally advanced disease. Where stage was reported as a basic number, only stages II and III were counted. For studies reporting stage as a basic category, only ‘regional’ was counted based on the Surveillance, Epidemiology, and End Results summary staging descriptions (Online Supplemental Appendix); this excludes patients with IB2 and IVA disease as these were grouped with stage IA/IB1 or IVB in the ‘localized’ and ‘distant’ groups, respectively. For studies reporting stage according to the FIGO classification (any version), stages IB2–IVA were counted. Estimates of the proportions of locally advanced cervical cancer were calculated by dividing the number of cases of locally advanced stages by the total cervical cancer population. To address large variations in the number of patients included in each study, a post hoc analysis evaluated the estimated proportions for each study according to data source (registry, multicenter, single center; online supplemental figure 2).

## RESULTS

### Search Results

**Figure 1:**
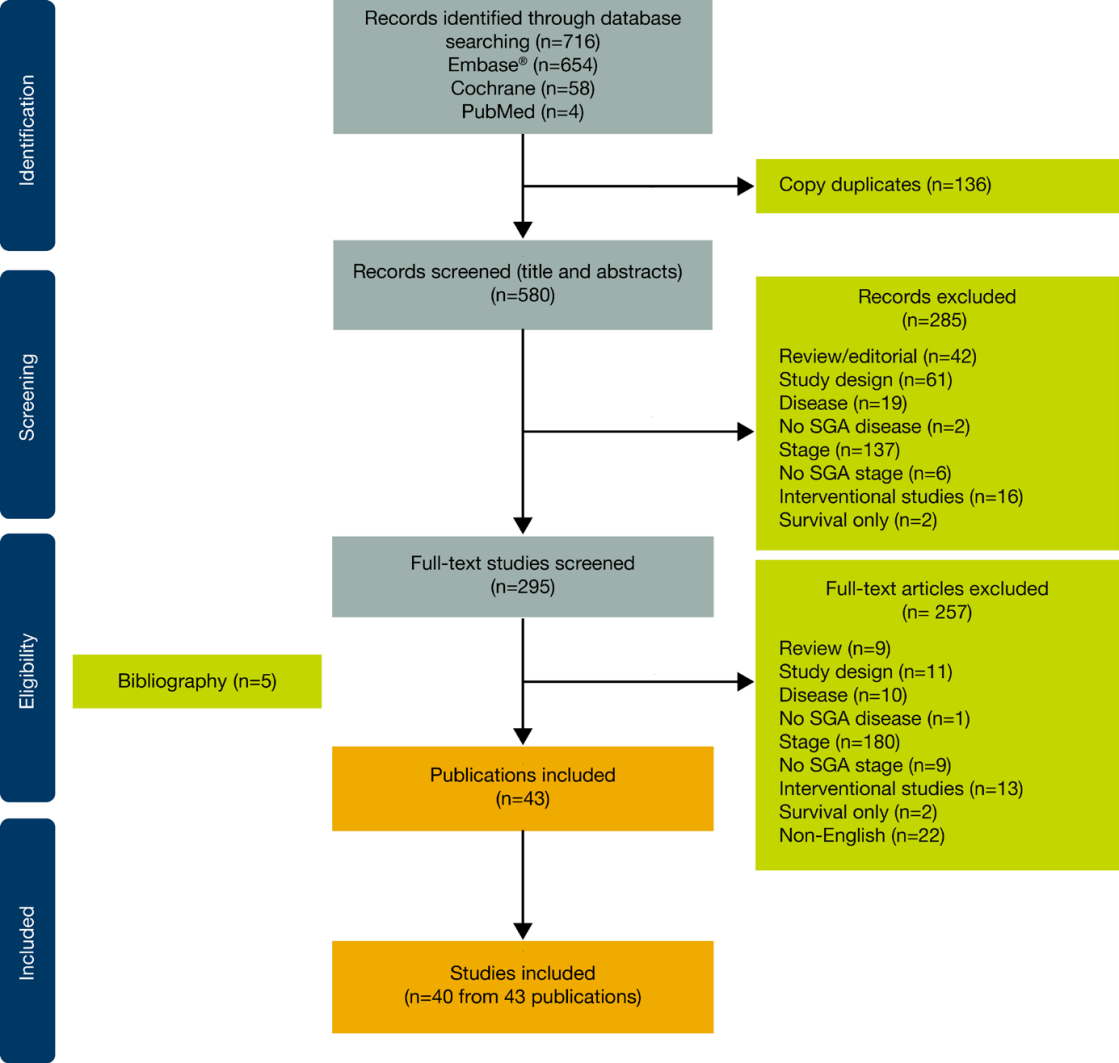
Preferred Reporting Items for Systematic reviews and Meta-Analysis (PRISMA) flow diagram. PRISMA guidelines were published in Page MJ, *et al*. The PRISMA 2020 statement: an updated guideline for reporting systematic reviews. *BMJ* 2021;372:n71. SGA, subgroup analysis.

In total, 716 references were identified through database searches; 580 of these were screened by title and abstract, resulting in 295 references that underwent full-text screening. Five references were identified through bibliography and gray literature searches and added to the final set. Figure 1 illustrates the selection process for the 43 publications (representing 40 studies) that were included in this systematic literature review.

### Study Characteristics

The 40 included studies represented 28 individual countries in North America, South America, Asia, Europe, Africa, and the Caribbean (online supplemental figure 1 and table 5). Across studies there was variation in population size, data source, time frame of data collection, and patient selection (online supplemental table 5). The total number of patients with cervical cancer ranged from 19 to 87 151. All but three studies used a retrospective cohort design; the remaining studies used a case–control,^4^ prospective observational,^5^ or longitudinal cohort design.^6^ Data sources were regional, national, or multinational cancer registries (29 studies (73%)) or obtained data from a single institution (seven studies (18%)), multiple institutions (two studies (5%)), or a healthcare database (two studies (5%)). Nine studies used data from the United States Surveillance, Epidemiology, and End Results database; variations in registries, data collection period, tumor staging criteria used, and populations assessed probably minimized overlap in patient data among these studies (online supplemental table 5). The median data collection period for all included studies was 13 years (range 1–47 years); 35 studies (88%) collected data between 1987 and 2017. One study did not provide dates of data capture.^7^

Twenty-nine studies (73%) included women with any stage of cervical cancer and no additional exclusion criteria on the population. Others limited analyses to patients with locally advanced stages of disease,^8 9^ patients ≥60 years,^4 10 11^ patients 19–30 years,^12–14^ or specific races/ethnicities.^15 16^ One study compared a population of pediatric and young adult cancer survivors subsequently diagnosed with cervical cancer after age 30 with women from the general population who were diagnosed with cervical cancer before age 56.^6^ All three types of cervical cancer staging (ie, basic number without further detail, basic category, and full FIGO classification) appeared in the included articles. Additionally, two studies reported disease as ‘locally advanced,’ ‘adjacent organs,’ and ‘regional lymph nodes’.^6 17^

### Proportions of Locally Advanced Cervical Cancer

**Figure 2:**
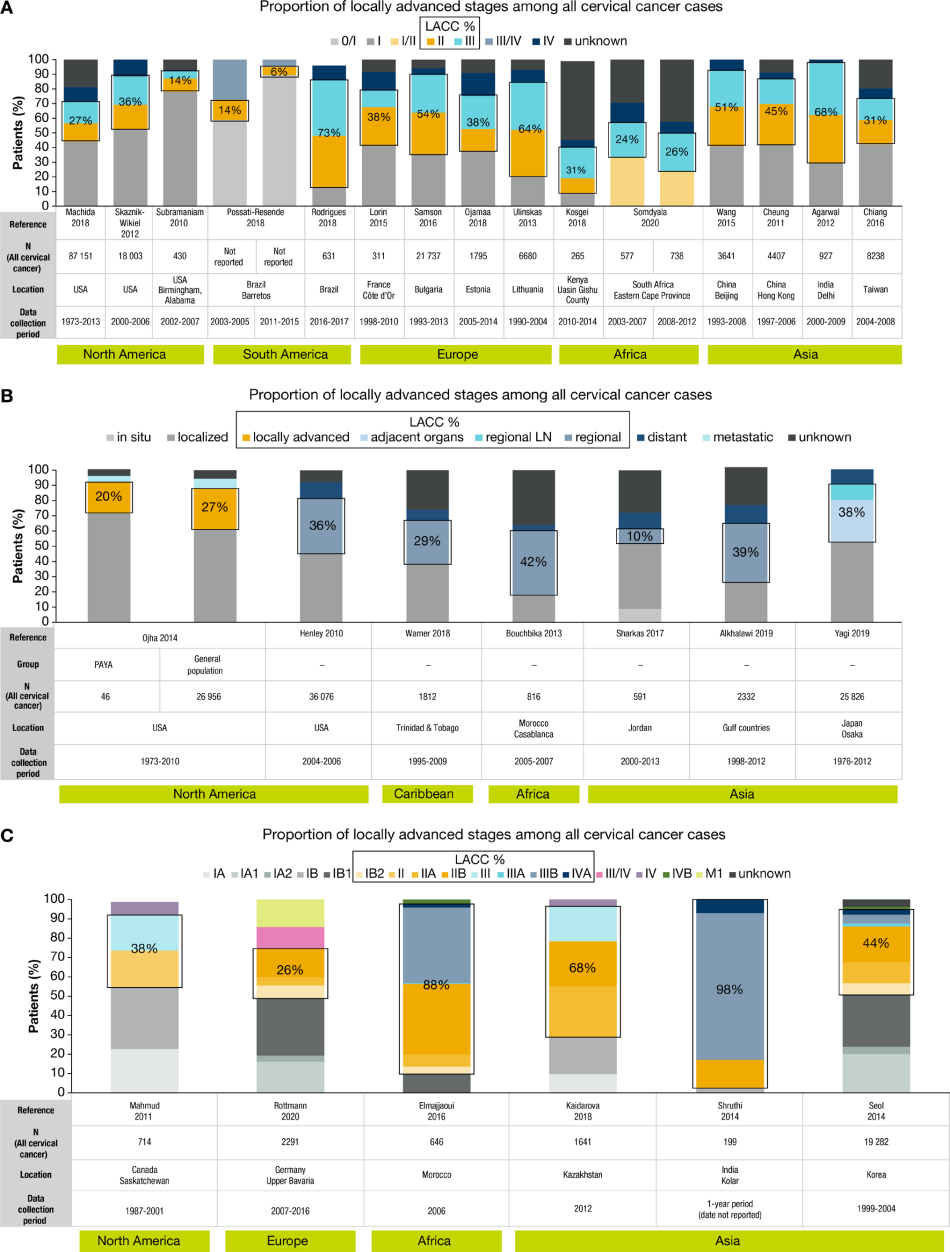
Estimated proportions of locally advanced cervical cancer among all patients with cervical cancer. (A) Studies reporting stage as a basic number without further detail (ie, I–IV).^5 19 21–23 26–28 31 33 46–50^ (B) Studies reporting stage as a basic category.^6 17 25 29 30 32 34^ (C) Studies reporting stage as full FIGO classification.^7 20 24 51–53^ FIGO, International Federation of Gynecology and Obstetrics; LACC, locally advanced cervical cancer; PAYA, pediatric and young adult.

Of the 38 studies (95%) that reported proportions of patients with locally advanced cervical cancer, nine excluded patients based on age, race, or disease stage.^4 8–10 12–16^ Figure 2 presents the proportion of patients by disease stage as reported in 28 of the 29 studies without excluded patients. The remaining study, Carmo et al, reported stage II or III disease among 68.3% of patients with cervical cancer at their center in Rio de Janeiro.^18^ Collectively, these 29 studies reported on 278 032 women with cervical cancer, of whom at least 99 471 had locally advanced disease according to our calculations.

The estimated median proportion of locally advanced cervical cancer among the 29 studies was 37.0% (range 5.6–97.5%; IQR 25.8–52.1%). Estimates of at least 50% were calculated for nine studies from Asia,^7 19–21^ Europe,^22 23^ Brazil,^5 18^ and Morocco.^24^ Estimates of ≤25% were calculated for six studies from Asia,^17 25^ the United States,^6 26^ Brazil,^27^ and South Africa.^28^ The three studies from Brazil had a wide range of estimated patients with locally advanced disease (6–73%).^5 18 27^ Eighteen studies reported the proportion of patients with unknown stage, with six reporting ≥25% unstaged.^25 28–32^ Overall, lower proportions of locally advanced disease were observed in North America versus Asia, Europe, and other regions, with Asia having the highest overall proportions (Figure 2).

Stage IB2 and/or IVA disease were grouped with other stages not considered locally advanced in 26/29 studies (90%) (Figure 2). Additionally, although the 29 studies reporting proportions of locally advanced disease among all patients with cervical cancer were a heterogenous group, each was given equal weight regardless of sample size, data source, or other factors that could influence the reported proportions. Analysis of proportions of locally advanced disease by data source further highlighted the heterogeneity of studies (online supplemental figure 2).

### Proportions of Locally Advanced Cervical Cancer by Age

Nine studies analyzed patients according to age group (Table 1).^4 8–10 12–14 18 33^ Litvinova et al reported that all ‘young women’ (age not disclosed) in Minsk City, Belarus, had locally advanced cervical cancer, primarily stage IIB–III.^14^ The proportion of locally advanced stages was low among women aged 19–29 years in Newfoundland, Canada (13.9%), and 19–30 years in Bradford, UK (5.3%); early-stage disease constituted the majority of cases.^12 13^ In Dublin, Ireland, and the United States, 49% and 44% of women with cervical cancer aged ≥60 and ≥70 years, respectively, had stage II–III disease.^4 10^

**Table 1:** Estimated proportions of locally advanced cervical cancer by patient age

Reference	Region	Location/data collection period	Age group, years	N	Percent with locally advanced cervical cancer (total)	Percent with locally advanced cervical cancer (by stage)
Studies of older women with cervical cancer						
McLean et al^4^ 2012	North America	USA 1992–2007	≥70, Medicare enrolled	734	44.4%	II, 20.8% III, 23.6%
Garry et al^10^ 2018	Europe	Ireland Dublin 2006–2015	≥60	119	49.0%	II, 28.0% III, 21.0%
Studies of young women with cervical cancer						
Popadiuk et al^12^ 2010	North America	Canada Newfoundland 1992–2008	19–29	37	13.9%	IIB, 9.3% IIIB, 2.3% IVA, 2.3%
Litvinova et al^14^ 2017	Europe	Belarus Minsk City 2012–2016	Young women	94	100%	IIB, 45.7% III, 52.2% IVA, 2.1%
Nathani et al^13^ 2012	Europe	UK Bradford 2007–2011	19–30	19	5.3%	III, 5.3%
Studies reporting proportions of age groups by stage of cervical cancer						
Tian et al*^9^ 2020	North America	USA 2010–2015	Squamous cell <40 40–49 50–59 60–69	3385 24.8% 28.5% 28.0% 18.7%	100%	–
			Adenocarcinoma <40 40–49 50–59 60–69	746 21.2% 33.9% 26.7% 18.2%	100%	–
Skaznik-Wikiel et al^33^ 2012	North America	USA 2000–2006	(All ages) ≥70	18 003 ~12%	Not calculable	IIIB, 58.8%
Garg et al^8^ 2011	North America	USA 1988–2005	Mean age, years 54 49	271 289	– –	IIA1 IIA2
Carmo et al^18^ 2011	South America	Brazil Rio de Janeiro 1999–2004	(All ages) <65 ≥65	3341	70.3%	II (n=1133) 79.9% 20.1%
			<65 ≥65			III (n=1216) 78.1% 21.9%

*The Tian 2020 study^9^ only included patients with locally advanced stages of cervical cancer and squamous cell or adenocarcinoma histology; patients with squamous cell and adenocarcinoma histology were separated for all analyses.

Four studies analyzed stage and age groups among patients with cervical cancer of all ages. In Brazil, age group distribution was significantly different between stage I, II, III, and IV (p<0.001): about 20% of patients with locally advanced disease were ≥65 years compared with about 11% of patients with stage I disease.^18^ In one study in the United States, the majority (78.6%) of patients aged <30 years were diagnosed with stage IA1, whereas most (58.8%) patients ≥70 years of age were diagnosed with stage IIIB disease.^33^ In an analysis of United States women with stage IIA cervical cancer, patients with IIA2 disease were younger than those with stage IIA1 disease (p=0.01).^8^ In a third United States study, the distribution of adenocarcinoma and squamous cell carcinoma varied by patient age. A higher proportion of patients with adenocarcinoma were 40–49 years old (33.9%) compared with the proportion of patients with squamous cell carcinoma (28.5%). In all other age groups, the proportions were similar or slightly higher for squamous cell carcinoma.^9^

### Proportions of Locally Advanced Cervical Cancer by Histology

Five studies analyzed tumor histology by disease stage (Table 2).^7–9 18 19^ Squamous cell carcinoma was the most common histologic type. Garg et al found no statistically significant difference in histology distribution between stages IIA1 and IIA2 cervical cancer.^8^ In Carmo et al, adenocarcinoma and adenosquamous carcinoma (plus other histologic types) were more frequent in stages I and II (p<0.001).^18^ Tian et al found significantly more patients with FIGO stages IIB–IVA in the squamous cell group compared with the adenocarcinoma group (p<0.001).^9^

**Table 2:** Comparisons of tumor histology among locally advanced stages of cervical cancer

Reference	Region	Location/data collection period	Locally advanced stage of cervical cancer	N	Histologic group
Garg et al*^8^ 2011	North America	USA 1988–2005	IIA1	271	SCC, 79.7% AC, 13.3% ASC, 7.0%
			IIA2	289	SCC, 83.0% AC, 12.8% ASC, 4.2%
Tian et al†^9^ 2020	North America	USA 2010–2015	IB2, 10.8% IIA1, 2.4% IIA2, 4.0% IIB, 21.6% IIIA, 2.2% IIIB, 54.3% IVA, 4.7%	3385	SCC
			IB2, 19.0% IIA1, 3.4% IIA2, 5.6% IIB, 21.4% IIIA, 2.0% IIIB, 46.4% IVA, 2.1%	746	AC
Carmo et al^18^ 2011	South America	Brazil Rio de Janeiro 1999–2004	II	1133	SCC, 83.3% AC, 12.9% ASC, 3.8%
			III	1216	SCC, 89.1% AC, 8.4% ASC, 2.5%
Agarwal et al^19^ 2012	Asia	India Delhi 2000–2009	II	306	SCC, 92.8% AC, 5.6% Other, 1.6%
			III	328	SCC, 95.4% AC, 4.3% Other, 0.3%
Shruthi et al^7^ 2014	Asia	India Kolar 1-year period (date not reported)	IIB	29	WDSCC, 17.2% MDSCC, 69.0% PDSCC, 13.8% AC, 0
			IIIB	151	WDSCC, 17.9% MDSCC, 57.6% PDSCC, 21.9% AC, 2.6%
			IVA	14	WDSCC, 0 MDSCC, 0 PDSCC, 100% AC, 0

*The Garg 2011 study^8^ only included patients with stage IIA cervical cancer, with analyses comparing IIA1 and IIA2.

†The Tian 2020 study^9^ only included patients with locally advanced stages of cervical cancer and squamous cell or adenocarcinoma histology; patients with squamous cell and adenocarcinoma histology were separated for all analyses.

AC, adenocarcinoma; ASC, adenosquamous carcinoma; MDSCC, moderately differentiated squamous cell carcinoma; PDSCC, poorly differentiated squamous cell carcinoma; SCC, squamous cell carcinoma; WDSCC, well-differentiated squamous cell carcinoma.

### Proportions of Locally Advanced Cervical Cancer by Race or Ethnicity

Five studies reported locally advanced disease according to racial or ethnic groups (Table 3).^8 9 15 16 18^ In the United States Pacific Northwest, American Indian/Alaskan Native women with cervical cancer had increased proportions of locally advanced disease compared with White women (33.9% vs 29.9%), though not statistically significant.^15^ Hou et al reported that 18.5% of Asian American women in the United States and 7.9% of White women had locally advanced disease (p<0.001).^16^ Garg et al found no significant difference in racial distribution between patients with stage IIA1 and IIA2 disease.^8^ In another study from the United States, the squamous cell carcinoma group included more Black women than the adenocarcinoma group (p<0.001).^9^ Among women from Rio de Janeiro, Brazil, no difference was observed in the distribution of cases among White and non-White patients by disease stage (p=0.168).^18^

**Table 3:** Comparisons of race or ethnicity among patients with locally advanced stages of cervical cancer

Reference	Region	Location / data collection period	Locally advanced stage of cervical cancer	N	Race or ethnic group
Garg et al*^8^ 2011	North America	USA 1988–2005	IIA1	271	Black, 10.7% White, 72.7% Other, 16.6%
			IIA2	289	Black, 10.4% White, 75.8% Other, 13.8%
Hou et al†^16^ 2019	North America	USA 1988–2011	II, 11.6% III, 6.9% Total LACC, 18.5%	3953	Asian American
			II, 4.8% III, 3.1% Total LACC, 7.9%	54 827	White
Bruegl et al†^15^ 2020	North America	USA Idaho, Oregon, & Washington 1996–2016	Regional, 33.9%	221	AI/AN
			Regional, 29.9%	7001	Non-Hispanic White
Tian et al‡^9^ 2020	North America	USA 2010–2015	Squamous cell, all LACC stages	3385	Black, 16.8% White, 73.1% Other, 10.1%
			Adenocarcinoma, all LACC stages	746	Black, 7.9% White, 79.9% Other, 12.2%
Carmo et al^18^ 2011	South America	Brazil Rio de Janeiro 1999–2004	II	1133	Non-White, 42.9% White, 57.1%
			III	1216	Non-White, 47.0% White, 53.0%

*The Garg 2011 study^8^ only included patients with stage IIA cervical cancer, with analyses comparing IIA1 and IIA2.

†The Hou 2019 study^16^ only included patients who were Asian American or White. The Bruegl 2020 study^15^ only included patients who were AI/AN or non-Hispanic White.

‡The Tian 2020 study^9^ only included patients with LACC stages of disease and squamous cell or adenocarcinoma histology; patients with squamous cell and adenocarcinoma histology were separated for all analyses.

AI/AN, American Indian/Alaskan Native; LACC, locally advanced cervical cancer.

### Incidence of Locally Advanced Cervical Cancer

Five studies reported incidence according to disease stage (Online Supplementary Table 6).^11 14 15 34 35^ In two studies from the United States, incidence rates for locally advanced cervical cancer were 2–4/100 000 women among different time frames.^15 34^ Another United States study reported that the incidence of stage I cervical cancer decreased by 2.4% per year (p<0.0001), whereas stage III disease increased by 2.0% per year (p=0.0002) in women aged ≥65 years during 1992–2007.^11^ A fourth United States study compared the incidence of localized and distant cervical cancer in urban and rural areas; neither stage was considered locally advanced in our analysis.^35^ In Minsk City, Belarus between 2012 and 2016, incidence of unresectable cervical cancer decreased from 3.8 to 1.9/100 000 female population for stage IIB and from 3.2 to 2.3/100 000 female population for stage III, but increased from 0.4 to 0.7/100 000 female population for stage IVA.^14^

## DISCUSSION

### Summary of Main Results

In this systematic literature review, we collected data from relevant observational studies around the world to provide an estimate of the proportion and incidence of locally advanced cervical cancer. Our searches identified 39 studies reporting proportions of, and five reporting incidence of, locally advanced cervical cancer. A median 37% of worldwide cervical cancer cases were locally advanced, with variation between geographic regions. Studies from North America and Asia reported lower and higher proportions, respectively, of locally advanced disease relative to other world regions; this might be due to differences in guidelines for screening, healthcare access, cultural influences on healthcare use, and limitations of data sources and methodologies used in data collection. Given the heterogeneity of studies and our conservative definitions of locally advanced disease, our findings probably underestimate the true proportions of locally advanced cervical cancer.

### Results in the Context of Published Literature

Although many publications report epidemiology of cervical cancer based on large registries, information by stage is rarely included.^1 36^ To our knowledge, this is the first systematic literature review to attempt to create a global picture of the epidemiology of locally advanced disease using observational studies. Variation in the proportion of locally advanced disease might reflect differences in healthcare access. Multiple studies have shown or projected that cervical cancer incidence and mortality is preventable if screening and human papillomavirus vaccination are used effectively.^37–40^ Implementation has been hindered by limitations in country-level resources and individual patient socioeconomic factors.^41–43^ In these populations, failure to detect pre-cancerous cervical lesions and invasive cervical cancer might lead to higher rates of locally advanced disease.

### Strengths and Weaknesses

Our study used a systematic approach to identify eligible studies and took a conservative approach toward defining locally advanced stages. However, several factors limit the extent of the conclusions. The heterogeneity of study characteristics and style of reporting disease stage were significant limitations. Population size, inclusion and exclusion criteria, data source, and time periods of data capture differed among studies. Owing to the descriptive nature of this review, these factors were not accounted for when estimating the proportion of locally advanced disease. Notably, Hispanic patients were not included in any study that reported the proportion of locally advanced disease according to ethnicity. While subgroup analyses among patients with locally advanced cervical cancer provided additional insight, no conclusions could be drawn. Similarly, no conclusions could be drawn about incidence due to the small number of studies. Generalizability of certain studies to a specific country or region should be cautiously considered, as many studies represented a specific locality or community.

A limitation of epidemiology studies in general is the influence of unknown or missing stage on prevalence and incidence estimates. Missing information is a recognized problem with population-based registries, and large observational studies might exclude patients with missing data from the analysis. When patients with missing data are included, the computational methods for handling this lack of information are inconsistently reported.^44^ The inability to account for patients with missing data might alter epidemiologic findings—as in a recent analysis of the association between missing data and overall survival in the National Cancer Database (United States), which found that specific types of missing information, including cancer stage and pathologic characteristics, were significantly associated with overall survival.^45^

The authors also acknowledge as a limitation that this analysis only included articles published through June 2020. Given that this extensive analysis of 10 years of literature yielded 40 studies, we anticipate a small number of additional studies published between June 2020 and August 2022 that would fit the systematic literature review criteria or substantially impact the results described.

### Implications for Practice and Future Research

The estimated global proportion of locally advanced cervical cancer reported here suggests a need for better prevention and screening practices for low-resource countries and underserved populations within high-resource countries. Additionally, the majority of epidemiology data captured in our systematic literature review did not define locally advanced stages in the same manner that clinicians typically use to determine optimal treatment. Only 12 studies provided detailed FIGO stage, which is commonly used to determine treatment in a clinical setting. Furthermore, owing to the retrospective nature of most epidemiology studies, staging data do not reflect the most recent criteria (ie, FIGO 2018, which is likely to assign more patients to locally advanced disease according to the new criteria for stage IIIC). Defining the locally advanced population and burden in different regions will allow clinicians to identify patients who might benefit from evolving therapeutic strategies.

## CONCLUSIONS

This systematic literature review highlights the high number of patients with locally advanced cervical cancer worldwide and disparities in epidemiology according to region, age, and race.

## Data Availability

Data are available upon reasonable request. The protocol for the systematic literature review, while unregistered, is readily available upon request to the corresponding author.
