# Supplementary material for: Proportions and incidence of locally advanced cervical cancer: a global systematic literature review
Source: Int J Gynecol Cancer. 2022 Oct 14;32(12):1531–9. doi: 10.1136/ijgc-2022-003801 (PMC9763192; doi:10.1136/ijgc-2022-003801)
Supplement: Supplementary data [file ijgc-2022-003801supp001.pdf]

SUPPLEMENTARY APPENDIX

Proportions and incidence of locally advanced cervical cancer: a global systematic literature review

Bradley J Monk,[1] David S P Tan,[2] José David Hernández Chagüi,[3] Jitender Takyar,[4] Michael J. Paskow,[3] Ana Tablante Nunes,[5] Eric Pujade-Lauraine[6]

Supplementary Methods

The search strategies shown below were created to support this locally advanced cervical cancer epidemiology systematic literature review as well as one focused on the natural history of locally advanced cervical cancer. Only the epidemiology publications are reported in this article. EMBASE, MEDLINE (PubMed), and Cochrane databases were searched using the search strategies below. Because some studies are not appropriately indexed in electronic databases, bibliographic searching and pearl growing techniques were used to identify any potentially relevant studies that were not captured by database searches.

Embase search strategy run on June 10, 2020

Search number	Query	Results
#1	'uterine cervix cancer'/syn	114 434
#2	'cervical tumor' OR 'cervical neoplasm' OR 'cervical tumour' OR 'cervical cancer'	67 038
#3	cervi* NEAR/5 (cancer* OR oncolog* OR neoplas* OR carcinom* OR malignan* OR tumor* OR tumour* OR mass* OR growth* OR cyst* OR adenocarcinom* OR squamous)	159 269
#4	#1 OR #2 OR #3	159 271
#5	'natural history'/exp OR 'natural history':ab,ti,kw OR 'natural course'	423 926
#6	'natural history study'	1649
#7	('observational' OR 'prospective' OR 'retrospective' OR 'cross-sectional' OR 'cross sectional' OR 'longitudinal') NEAR/3 ('study' OR 'studies' OR analys*)	2 513 311
#8	#5 AND #7	18 090
#9	'disease course':ab,ti,kw OR 'clinical course' OR ('natural history' NEAR/2 prognos*)	113 467
#10	'inception cohort' OR 'disease exacerbation'/syn OR 'disease progression' OR 'outcome assessment':ab,ti,kw	246 377
#11	#5 OR #6 OR #8 OR #9 OR #10	770 081
#12	#4 AND #11	3494
#13	'locally advanced' OR 'local advanced' OR (local* NEAR/2 'advanced') OR 'stage one' OR 'stage two' OR 'stage three' OR 'stage four' OR 'stage ib2' OR 'stage iib'	103 225

	OR 'stage iiia' OR 'stage iiib' OR 'stage iva' OR 'stage 1b2' OR 'stage 2b' OR 'stage 3a' OR 'stage 3b' OR 'stage 4a' OR ('stage' AND ('ib2' OR 'iib' OR 'iiia' OR 'iiib' OR 'iva' OR '1b2' OR '2b' OR '3a' OR '3b' OR '4a')) OR ('stage' NEAR/2 ('ib2' OR 'iib' OR 'iiia' OR 'iiib' OR 'iva' OR '1b2' OR '2b' OR '3a' OR '3b' OR '4a')) OR 'non-metastatic' OR 'non metastatic' OR 'lacc'	
#14	'epidemiology':ab,ti OR epidemi*:ab,ti OR 'incidence':ab,ti OR inciden*:ab,ti OR 'prevalence':ab,ti OR prevalen*:ab,ti OR 'seasonal variation':ab,ti OR 'mortality':ab,ti OR 'morbidity':ab,ti OR complication*:ab,ti	4 471 505
#15	epidemiolog* NEAR/2 ('study' OR 'studies' OR analys*)	136 119
#16	#14 OR #15	4 476 280
#17	#13 AND #16	26 494
#18	#4 AND #17	2549
#19	#12 OR #18	5995
#20	#19 AND ([conference abstract]/lim OR [conference paper]/lim OR [conference review]/lim OR [letter]/lim OR [note]/lim OR [review]/lim)	2540
#21	#19 AND [animals]/lim	126
#22	#20 OR #21	2598
#23	#19 NOT #22	3397
#24	#23 AND [2010-2020]/py	1671
#25	#24 AND [english]/lim	1551

PubMED search strategy run on June 10, 2020

Search number	Query	Results
#1	Search: 'uterine cervix cancer'[MeSH Terms]	74 490
#2	Search: “cervical tumor” OR “cervical neoplasm” OR “cervical tumour” OR “cervical cancer”	47 147
#3	Search: cervi* AND (cancer* OR oncolog* OR neoplas* OR carcinom* OR malignan* OR tumor* OR tumour* OR mass* OR growth* OR cyst* OR adenocarcinom* OR squamous)	161 507
#4	Search: #1 OR #2 OR #3	161 507
#5	Search: ('natural history'[MeSH Terms]) OR ('natural history'[Title/Abstract]) OR 'natural course'	84 236
#6	Search: "natural history study"	776
#7	Search: (“observational” OR “prospective” OR “retrospective” OR “cross-sectional” OR “cross sectional” OR “longitudinal”) AND (“study” OR “studies” OR analys*)	2 332 275
#8	Search: #5 AND #7	17 334
#9	Search: “disease course”:[Title/Abstract] OR “clinical course” OR (“natural history” AND prognos*)	81 583
#10	Search: ((“inception cohort”) OR (“disease exacerbation”[MeSH Terms]) OR “disease progression”) OR (“outcome assessment”[MeSH Terms])	218 726
#11	Search: #5 OR #6 OR #8 OR #9 OR #10	360 274
#12	Search: #4 AND #11	4419
#13	Search: “locally advanced” OR “local advanced” OR (local* AND “advanced”) OR “stage one” OR “stage two” OR “stage three” OR “stage four” OR “stage ib2” OR “stage iib” OR “stage iiia” OR “stage iiib” OR “stage iva” OR “stage 1b2” OR “stage 2b” OR “stage 3a” OR “stage 3b” OR “stage 4a” OR (“stage” AND (“ib2” OR “iib” OR “iiia” OR “iiib” OR “iva” OR “1b2” OR “2b” OR “3a” OR	100 166

	"3b" OR "4a")) OR ("stage" AND ("ib2" OR "iib" OR "iiia" OR "iiib" OR "iva" OR "1b2" OR "2b" OR "3a" OR "3b" OR "4a")) OR "non-metastatic" OR "non metastatic" OR "lacc"	
#14	Search: "epidemiology"[Title/Abstract]OR epidemi*[Title/Abstract]OR "incidence"[Title/Abstract]OR inciden*[Title/Abstract]OR "prevalence"[Title/Abstract]OR prevalen*[Title/Abstract]OR "seasonal variation"[Title/Abstract]OR "mortality"[Title/Abstract]OR "morbidity"[Title/Abstract]OR complication*[Title/Abstract]	3 291 414
#15	Search: epidemiolog* AND (study OR studies OR analys*)	1 520 332
#16	Search: #14 OR #15	3 926 004
#17	Search: #13 AND #16	25 044
#18	Search: #4 AND #17	2117
#19	Search: #12 OR #18	6471
#20	Search: (#19 AND (inprocess[sb] OR pubstatusaheadofprint))	131

Cochrane search strategy run on June 10, 2020

#1	MeSH descriptor: [Uterine Cervical Neoplasms] explode all trees	MeSH	2020
#2	"natural history" OR "natural history":ab,ti,kw OR "natural course"	Limits	3135
#3	"natural history study"	Limits	83
#4	("observational" OR "prospective" OR "retrospective" OR "cross-sectional" OR "cross sectional" OR "longitudinal") NEAR/3 ("study" OR "studies" OR analys*)	Limits	214449
#5	#2 AND #4	Limits	1023
#6	"disease course":ab,ti,kw OR "clinical course" OR ('natural history' NEAR/2 prognos*)	Limits	14110
#7	"inception cohort" OR "disease exacerbation" OR "disease progression" OR "outcome assessment":ab,ti,kw	Limits	77386
#8	#2 OR #3 OR #5 OR #6 OR #7	Limits	90000
#9	#1 AND #8	Limits	147
#10	OR "stage iiia" OR "stage iiib" OR "stage iva" OR "stage 1b2" OR "stage 2b" OR "stage 3a" OR "stage 3b" OR "stage 4a" OR ("stage" AND ("ib2" OR "iib" OR "iiia" OR "iiib" OR "iva" OR "1b2" OR "2b" OR "3a" OR "3b" OR "4a")) OR ("stage" NEAR/2 ("ib2" OR "iib" OR "iiia" OR "iiib" OR "iva" OR "1b2" OR "2b" OR "3a" OR "3b" OR "4a")) OR "non-metastatic" OR "non metastatic" OR "lacc"	Limits	17625
#11	("epidemiology" OR epidemi* OR "incidence" OR inciden* OR "prevalence" OR prevalen* OR "seasonal variation" OR "mortality" OR "morbidity" OR complication*):ab,ti	Limits	270955
#12	epidemiolog* NEAR/2 ('study' OR 'studies' OR analys*)	Limits	6771
#13	#11 OR #12	Limits	272600
#14	#10 AND #13	Limits	3863
#15	#14 AND #1	Limits	102
#16	#15 OR #9	Limits	103

with Publication Year from 2010 to 2020, in Trials

The following conferences were also searched for relevant abstracts from meetings held between January 2017 and June 2020: American Society of Clinical Oncology, European Society for Medical Oncology, European Society of Gynaecological Oncology, Society of Gynecologic Oncology, American Association for Cancer Research, International Society for Pharmacoeconomics and Outcomes Research, International Gynecologic Cancer Society. The publication timeframe for conference searching was limited to the previous 3 years

based on the assumption that research presented at conferences is usually published within 3–4 years as a full-text article or indexed in different biomedical literature databases as a conference paper, conference review, etc.

Inclusion and exclusion criteria used to identify relevant studies are shown in the table below.

Parameter	Inclusion/exclusion criteria
Study design	• Retrospective observational study<br>• Prospective observational study<br>• Case-control studies<br>• Surveys and cross-sectional studies<br>• Registry/database studies<br>• Excluded: controlled trials (randomized controlled trial, non-randomized controlled study, or single-arm study)
Population	• Adult population (aged ≥18 years)<br>• Any race<br>• Locally advanced cervical cancer: stages IB2-IVA per any version of the FIGO staging criteria<br>• Excluded: studies that only include patients with early-stage or metastatic cervical cancer
Line of therapy	• Not restricted<br>• Studies of patients with locally advanced cervical cancer (both untreated and treated)
Countries	• Not restricted
Language	• English[a]
Time-frame	• 2010–2020
Data reported	• Proportion of patients with cervical cancer by disease stage

• Incidence of cervical cancer by disease stage

[a]English language was a criterion from the beginning of the systematic literature review process and was used as an exclusion criterion in database search queries.

FIGO, International Federation of Gynecology and Obstetrics.

Data extraction

The following information was extracted from the final set of published reports, where available: study details (sample size, inclusion/exclusion criteria, disease stage, stage classification criteria, treatment details, study limitations, time-frame of data collection, data source, location), patient demographics (age, race/ethnicity), clinical characteristics (histology, prior therapy), the proportion of patients with locally advanced stages of cervical cancer, prevalence (rate, odds ratio, risk ratio), and incidence (rate, risk ratio).

Calculation of the Proportion of Locally Advanced Cervical Cancer

The Surveillance, Epidemiology, and End Results summary stage categorizes the extent of cancer spread in a basic set of criteria. In the past, this classification system has also been referred to as General Stage, California Stage, historic stage, and Surveillance, Epidemiology, and End Results Stage. Summary stage uses all information available via medical records (ie, both clinical and pathologic documentation). Below are the criteria as per the most recent version (v2.0) published in 2020; however, studies included in the systematic literature review may have used older versions of the criteria. A summary of changes between the last available version (v1.7) and version 2.0 is available at https://seer.cancer.gov/tools/ssm/change-log.pdf. The 2020 criteria were used to determine which SEER Summary stages were equivalent to FIGO stage IB2-IVA.

2018 Surveillance, Epidemiology, and End Results summary staging criteria for cervical cancer[1]

Code	Stage	Definition
**0**	**In situ**	Noninvasive, intraepithelial lesions. Includes cancer in situ with endocervical gland involvement, cervical intraepithelial neoplasia Grade III, preinvasive.
**1**	**Localized**	Clinically visible lesion (macroscopic), including superficial invasion.

		Confined to cervix uteri or uterus NOS, except corpus uteri NOS, including if not clinically visible or unknown if clinically visible.<br>Measured stromal invasion less than 5 mm from the base of the epithelium AND horizontal spread of 7.0 mm or less.<br>Includes FIGO stage IA1, IA2, IA NOS, IB1, IB2, IB NOS, I NOS.
**2**	**Regional** (direct extension)	Extension to the bladder wall; bladder NOS excluding mucosa; bullous edema of bladder mucosa; confined to corpus uteri, size, depth and horizontal spread unknown; corpus uteri NOS; Cul de sac (rectouterine pouch); fallopian tube(s); "frozen pelvis" (clinically described); hydronephrosis or nonfunctioning kidney; invasion beyond uterus NOS; ligament(s) (broad, cardinal, uterosacral); ovary/ovaries; parametrial (paracervical soft tissue) invasion; pelvic wall(s); rectal wall; rectum NOS excluding mucosa; upper two-thirds of vagina including fornices; ureter (intra- and extramural); urethra; vagina (lower third [not extending into pelvic wall], NOS); vaginal wall NOS; vulva.<br>Includes FIGO stage IIA, IIB, II NOS, IIIA, IIIB, III NOS.
**3**	**Regional** (lymph node involvement only)	Localized tumor WITH regional lymph node involvement.<br>Involvement of the following types of lymph nodes: para-aortic, iliac NOS, paracervical, parametrial, sacral NOS, regional NOS.<br>Includes FIGO stages IIIC1, IIIC2, IIIC NOS.
**4**	**Regional** (both direct extension and regional lymph nodes involved)	Any combination of codes 2 and 3 above.
**7**	**Distant** (sites or lymph nodes)	Cervical cancer that has metastasized. Includes bladder mucosa, rectal mucosa, sigmoid colon, small intestine, inguinal (femoral) lymph node, mediastinal lymph node, scalene lymph node, supraclavicular lymph node; or cancers labeled as carcinomatosis or distant metastasis with or without distant lymph nodes.<br>Includes FIGO stage IVA, IVB, IV NOS.
**9**	**Unknown**	Unknown if extension or metastasis.

FIGO, International Federation of Gynecology and Obstetrics; NOS, not otherwise specified.

Monk BJ, *et al. Int J Gynecol Cancer* 2022; 32:1531–1539. doi: 10.1136/ijgc-2022-003801

Supplementary Fig 1. Countries represented by the studies included in the systematic literature review.

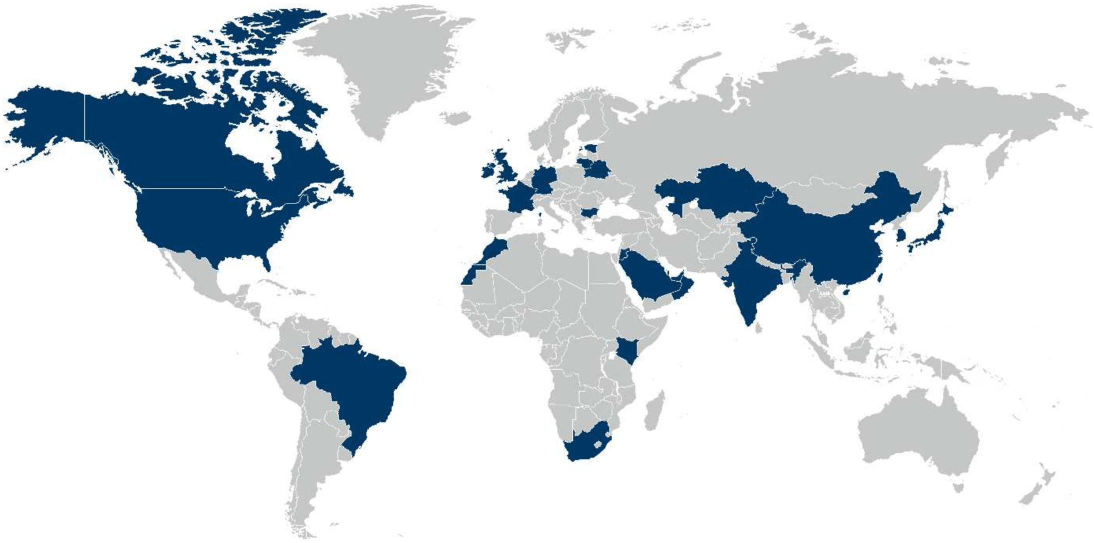

Included countries are the United States, Canada, China, India, Japan, Jordan, collective Gulf countries [Saudi Arabia, United Arab Emirates, Qatar, Oman, Kuwait, Bahrain], Kazakhstan, Korea, Taiwan, Belarus, Bulgaria, Estonia, France, Germany, Ireland, Lithuania, the United Kingdom, Morocco, Kenya, South Africa, Brazil, and Trinidad & Tobago.

Monk BJ, *et al. Int J Gynecol Cancer* 2022; 32:1531–1539. doi: 10.1136/ijgc-2022-003801

Supplementary Fig 2. Estimated proportion of locally advanced cervical cancer by type of data source

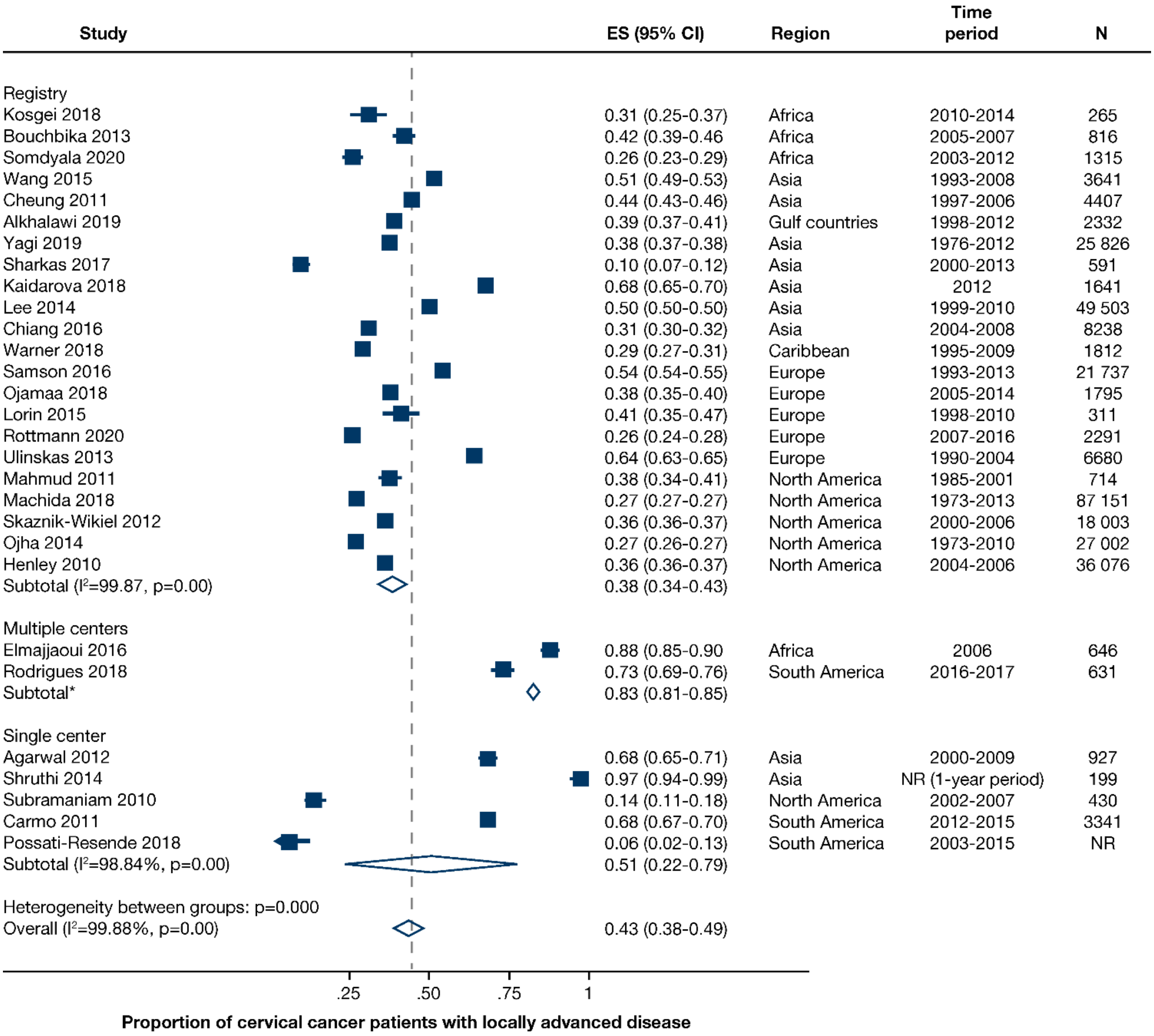

Estimated proportion for each study (ES) and the 95% confidence intervals are plotted according to data source (registry, multicenter institution, or single institution). Overlapping timeframes and duplicate data from the same study have been removed. Red triangles represent the range of the subtotal estimated proportion, and the red dashed line represented the overall estimated proportion of locally advanced cervical cancer from this dataset. Heterogeneity of studies is reflected in the I^2 value; a score of >60% = high heterogeneity. Single center studies provided the most unreliable data with the largest variance (estimated range, 6–97%). N indicates the total number of women with cervical cancer. NR, not reported.

Monk BJ, *et al. Int J Gynecol Cancer* 2022; 32:1531–1539. doi: 10.1136/ijgc-2022-003801

Supplementary Table 1. Strengthening the Reporting of Observational studies in Epidemiology checklist items

Section	Item No	Recommendation
**Title and abstract**	1	(*a*) Indicate the study's design with a commonly used term in the title or the abstract
		(*b*) Provide in the abstract an informative and balanced summary of what was done and what was found
**Introduction**		
Background/rationale	2	Explain the scientific background and rationale for the investigation being reported
Objectives	3	State specific objectives, including any prespecified hypotheses
**Methods**		
Study design	4	Present key elements of study design early in the paper
Setting	5	Describe the setting, locations, and relevant dates, including periods of recruitment, exposure, follow-up, and data collection
Participants	6	(*a*) *Cohort study*—Give the eligibility criteria, and the sources and methods of selection of participants. Describe methods of follow-up<br>*Case-control study*—Give the eligibility criteria, and the sources and methods of case ascertainment and control selection. Give the rationale for the choice of cases and controls

Monk BJ, *et al. Int J Gynecol Cancer* 2022; 32:1531–1539. doi: 10.1136/ijgc-2022-003801

		Cross-sectional study—Give the eligibility criteria, and the sources and methods of selection of participants
		(*b*) *Cohort study*—For matched studies, give matching criteria and number of exposed and unexposed<br>*Case-control study*—For matched studies, give matching criteria and the number of controls per case
Variables	7	Clearly define all outcomes, exposures, predictors, potential confounders, and effect modifiers. Give diagnostic criteria, if applicable
Data sources/ measurement	8*	For each variable of interest, give sources of data and details of methods of assessment (measurement). Describe comparability of assessment methods if there is more than one group
Bias	9	Describe any efforts to address potential sources of bias
Study size	10	Explain how the study size was arrived at
Quantitative variables	11	Explain how quantitative variables were handled in the analyses. If applicable, describe which groupings were chosen and why
Statistical methods	12	(*a*) Describe all statistical methods, including those used to control for confounding
		(*b*) Describe any methods used to examine subgroups and interactions
		(*c*) Explain how missing data were addressed

		(*d*) *Cohort study*—If applicable, explain how loss to follow-up was addressed<br>*Case-control study*—If applicable, explain how matching of cases and controls was addressed<br>*Cross-sectional study*—If applicable, describe analytical methods taking account of sampling strategy
		(*e*) Describe any sensitivity analyses
**Results**		
Participants	13*	(a) Report numbers of individuals at each stage of study—eg numbers potentially eligible, examined for eligibility, confirmed eligible, included in the study, completing follow-up, and analysed
		(b) Give reasons for non-participation at each stage
		(c) Consider use of a flow diagram
Descriptive data	14*	(a) Give characteristics of study participants (eg demographic, clinical, social) and information on exposures and potential confounders
		(b) Indicate number of participants with missing data for each variable of interest
		(c) *Cohort study*—Summarise follow-up time (eg, average and total amount)
Outcome data	15*	*Cohort study*—Report numbers of outcome events or summary measures over time
		Case-control study—Report numbers in each exposure category, or summary measures of

		exposure
		Cross-sectional study—Report numbers of outcome events or summary measures
Main results	16	(*a*) Give unadjusted estimates and, if applicable, confounder-adjusted estimates and their precision (eg, 95% confidence interval). Make clear which confounders were adjusted for and why they were included
		(*b*) Report category boundaries when continuous variables were categorized
		(*c*) If relevant, consider translating estimates of relative risk into absolute risk for a meaningful time period
Other analyses	17	Report other analyses done—eg, analyses of subgroups and interactions, and sensitivity analyses
**Discussion**		
Key results	18	Summarise key results with reference to study objectives
Limitations	19	Discuss limitations of the study, taking into account sources of potential bias or imprecision. Discuss both direction and magnitude of any potential bias
Interpretation	20	Give a cautious overall interpretation of results considering objectives, limitations, multiplicity of analyses, results from similar studies, and other relevant evidence
Generalisability	21	Discuss the generalisability (external validity) of the study results

Monk BJ, *et al. Int J Gynecol Cancer* 2022; 32:1531–1539. doi: 10.1136/ijgc-2022-003801

**Other information**		
Funding	22	Give the source of funding and the role of the funders for the present study and, if applicable, for the original study on which the present article is based

*Information should be given separately for cases and controls in case-control studies and, if applicable, for exposed and unexposed groups in cohort and cross-sectional studies.

Items as reported on the Strengthening the Reporting of Observational studies in Epidemiology website (https://www.equator-network.org/reporting-guidelines/strobe/).

Monk BJ, *et al. Int J Gynecol Cancer* 2022; 32:1531–1539. doi: 10.1136/ijgc-2022-003801

Supplementary Table 2. Strengthening the Reporting of Observational studies in Epidemiology – Abstract version – checklist items

Item	Recommendation
**Title**	Indicate the study's design with a commonly used term in the title (e.g cohort, case-control, cross sectional)
**Authors**	Contact details for the corresponding author
**Study design**	Description of the study design (e.g cohort, case-control, cross sectional)
**Objective**	Specific objectives or hypothesis
**Methods**	
Setting	Description of setting, follow-up dates or dates at which the outcome events occurred or at which the outcomes were present, as well as any points or ranges on other time scales for the outcomes (e.g., prevalence at age 18, 1998-2007).
Participants	*Cohort study*—Give the most important eligibility criteria, and the most important sources and methods of selection of participants. Describe briefly the methods of follow-up<br>*Case-control study*—Give the major eligibility criteria, and the major sources and methods of case ascertainment and control selection<br>*Cross-sectional study*—Give the eligibility criteria, and the major sources and methods of selection of participants
	Cohort study—For matched studies, give matching and number of exposed and

	unexposed
	Case-control study—For matched studies, give matching criteria and the number of controls per case
Variables	Clearly define primary outcome for this report.
Statistical methods	Describe statistical methods, including those used to control for confounding
**Results**	
Participants	Report Number of participants at the beginning and end of the study
Main results	Report estimates of associations. If relevant, consider translating estimates of relative risk into absolute risk for a meaningful time period
	Report appropriate measures of variability and uncertainty (e.g., odds ratios with confidence intervals)
**Conclusions**	General interpretation of study results

Checklist items were obtained from the EQUATOR network website: https://www.equator-network.org/reporting-guidelines/strobe-abstracts/.

Monk BJ, *et al. Int J Gynecol Cancer* 2022; 32:1531–1539. doi: 10.1136/ijgc-2022-003801

Supplementary Table 3. Strengthening the Reporting of Observational studies in Epidemiology checklist for included studies[2-30]

Study name	Title & abstract		Intro-duction		Methods														Results											Discussion				Other infor-mation
	1a	1b	2	3	4	5	6a	6b	7	8	9	10	11	12a	12b	12c	12d	12e	13a	13b	13c	14a	14b	14c	15	16a	16b	16c	17	18	19	20	21	22
Henley 2010	Y	Y	Y	Y	Y	Y	Y	Y	Y	Y	N	Y	Y	Y	Y	Y	N	N	Y	Y	N	Y	Y	N	Y	Y	Y	N	N	Y	Y	Y	N	N
Garg 2011	Y	Y	Y	Y	Y	Y	Y	Y	Y	Y	N	Y	Y	Y	Y	N	N	N	Y	N	N	Y	N	N	Y	Y	Y	Y	N	Y	Y	Y	N	N
Skaznik-Wikiel 2012	Y	Y	Y	Y	Y	Y	Y	Y	Y	Y	N	Y	Y	Y	Y	N	N	N	Y	N	N	Y	Y	N	Y	Y	Y	N	N	Y	Y	Y	N	N
Ojha 2014	Y	Y	Y	Y	Y	Y	Y	Y	Y	Y	Y	Y	Y	N	N	N	N	N	Y	N	N	Y	Y	Y	Y	Y	Y	N	N	Y	Y	Y	N	N
Machida 2018	Y	Y	Y	Y	Y	Y	Y	Y	Y	Y	N	Y	Y	Y	Y	Y	N	Y	Y	Y	N	Y	Y	N	Y	Y	Y	Y	Y	Y	Y	Y	N	Y
Zahnd 2018	Y	Y	Y	Y	Y	Y	Y	Y	Y	Y	N	Y	Y	Y	Y	N	N	N	Y	N	N	Y	Y	Y	Y	Y	Y	Y	N	Y	Y	N	N	N
Hou 2019	Y	Y	Y	Y	Y	Y	Y	Y	Y	Y	N	Y	Y	Y	Y	Y	N	N	Y	Y	Y	Y	Y	Y	Y	Y	Y	Y	N	Y	Y	Y	N	N
Bruegl 2020	Y	Y	Y	Y	Y	Y	Y	Y	Y	Y	N	Y	Y	Y	Y	Y	N	N	Y	Y	N	Y	Y	N	Y	Y	Y	Y	Y	Y	Y	Y	N	Y
Tian 2020	Y	Y	Y	Y	Y	Y	Y	Y	Y	Y	N	Y	Y	Y	Y	N	N	N	Y	N	N	Y	N	N	Y	Y	Y	N	Y	Y	Y	Y	N	Y
Mahmud 2011	Y	Y	Y	Y	Y	Y	Y	Y	Y	Y	N	Y	Y	Y	Y	N	N	N	Y	N	N	Y	N	N	Y	Y	N	N	N	Y	Y	Y	N	Y
Carmo 2011	Y	Y	Y	Y	Y	Y	Y	Y	Y	Y	Y	Y	Y	Y	Y	Y	N	N	Y	N	N	Y	Y	Y	Y	Y	Y	N	Y	Y	Y	Y	N	N
Possati-Resende 2018	Y	Y	Y	Y	Y	Y	Y	N	Y	Y	N	N	N	N	N	N	N	N	N	N	Y	N	N	Y	Y	N	N	N	N	Y	Y	Y	N	N
Warner 2018	Y	Y	Y	Y	Y	Y	Y	Y	Y	Y	N	Y	Y	Y	N	N	N	N	Y	N	Y	Y	Y	N	Y	N	N	N	N	Y	Y	Y	Y	Y
Lorin 2015	Y	Y	Y	Y	Y	Y	Y	Y	Y	Y	Y	Y	Y	Y	Y	Y	N	N	N	N	N	Y	Y	Y	Y	Y	Y	Y	N	Y	N	N	Y	N
Samson 2016	Y	Y	Y	Y	Y	Y	Y	Y	Y	Y	N	Y	Y	Y	Y	N	N	N	Y	N	N	Y	N	N	Y	N	N	N	N	Y	Y	Y	N	Y
Ojamaa 2018	Y	Y	Y	Y	Y	Y	Y	Y	Y	Y	N	Y	Y	Y	Y	N	N	N	Y	N	N	Y	N	N	Y	Y	Y	N	N	Y	Y	Y	N	Y
Ulinskas 2013	Y	Y	Y	Y	Y	Y	Y	Y	Y	Y	N	Y	Y	Y	Y	N	N	N	N	N	N	Y	Y	Y	Y	Y	Y	Y	N	Y	N	N	N	Y
Bouchbika 2013	Y	Y	Y	Y	Y	Y	Y	Y	Y	Y	N	N	Y	Y	Y	N	N	N	Y	N	N	Y	Y	N	Y	Y	Y	N	Y	Y	Y	Y	Y	N

Elmajjaoui 2016	Y	Y	Y	Y	Y	Y	Y	Y	Y	Y	N	Y	Y	Y	Y	N	N	N	Y	N	Y	Y	N	Y	Y	N	N	N	N	Y	N	N	Y	N
Somdyala 2020	Y	Y	Y	Y	Y	Y	Y	Y	Y	Y	N	Y	Y	Y	Y	Y	N	N	Y	N	N	Y	Y	N	Y	Y	Y	N	Y	Y	Y	Y	N	Y
Sharkas 2017	Y	Y	Y	Y	Y	Y	Y	N	Y	Y	N	Y	Y	Y	N	N	N	N	Y	N	N	Y	Y	N	Y	N	N	N	N	Y	N	N	N	N
Alkhalawi 2019	Y	Y	Y	Y	Y	Y	Y	Y	Y	Y	N	Y	Y	Y	Y	N	N	N	Y	N	N	Y	N	N	Y	N	N	N	N	Y	N	N	N	N
Agarwal 2012	Y	Y	Y	Y	Y	Y	Y	Y	Y	Y	N	Y	Y	Y	Y	N	N	N	Y	N	N	Y	N	N	Y	N	N	N	N	Y	N	N	N	N
Shruthi 2014	Y	Y	Y	Y	Y	Y	Y	Y	Y	Y	N	Y	Y	N	Y	N	N	N	Y	N	N	Y	N	N	Y	N	N	N	N	Y	N	N	N	N
Wang 2015	Y	Y	Y	Y	Y	Y	Y	Y	Y	Y	N	Y	Y	Y	Y	N	N	N	Y	N	Y	Y	N	N	Y	Y	Y	N	N	Y	N	Y	N	Y
Cheung 2011	Y	Y	Y	Y	Y	Y	Y	Y	Y	Y	N	Y	Y	Y	Y	N	N	N	Y	N	N	Y	Y	Y	Y	Y	Y	N	Y	Y	N	N	N	N
Yagi 2019	Y	Y	Y	Y	Y	Y	Y	Y	Y	Y	N	Y	Y	Y	Y	Y	N	N	Y	N	N	Y	N	N	Y	Y	Y	N	N	Y	Y	Y	N	N
Seol 2014	Y	N	Y	Y	Y	Y	Y	N	Y	Y	N	Y	Y	Y	Y	N	N	N	Y	N	N	N	Y	Y	N	Y	N	N	Y	Y	Y	Y	Y	Y
Chiang 2016	Y	Y	Y	Y	Y	Y	Y	Y	Y	Y	N	N	Y	Y	Y	N	N	N	N	N	N	N	N	Y	Y	Y	N	N	Y	Y	Y	N	N	Y

Checklist items are explained in Online Supplementary Table 1.

Supplementary Table 4. Strengthening the Reporting of Observational studies in Epidemiology – Abstract version - checklist for included studies [31-41]

Study Name	Title	Authors	Study Design	Objective	Methods				Results - Participants	Main results	Conclusions
					Setting	Participants	Variables	Statistics			
Subramaniam 2010	N	Y	Y	Y	Y	Y	Y	Y	Y	N	Y
McLean 2012 (Int J Gyn Can)	N	Y	N	Y	Y	Y	Y	Y	Y	N	Y
McLean 2012 (Gyn Oncol)	N	Y	Y	Y	Y	Y	Y	Y	Y	Y	Y
Popadiuk 2010	N	N	N	Y	Y	Y	N	N	Y	N	Y
Rodrigues 2018	N	Y	Y	Y	Y	Y	N	Y	Y	Y	Y
Nathani 2012	N	Y	N	Y	Y	Y	Y	N	Y	N	Y
Garry 2018	N	Y	Y	Y	Y	Y	N	N	Y	N	Y
Rottmann 2020	Y	Y	Y	Y	Y	Y	Y	Y	Y	Y	Y
Litvinova 2017	N	Y	N	Y	Y	Y	Y	N	Y	N	N
Kosgei 2018	N	Y	N	Y	Y	Y	Y	N	Y	N	Y
Kaidarova 2018	N	Y	N	Y	Y	Y	Y	Y	Y	N	Y

Checklist items are explained in Online Supplementary Table 2.

Monk BJ, *et al. Int J Gynecol Cancer* 2022; 32:1531–1539. doi: 10.1136/ijgc-2022-003801

Supplementary Table 5. Study characteristics

Study / publication type	Location	Study design & data source type	Time-frame	Data source	Classification criteria[a]	Stages of cervical cancer included	Population	Total patients with cervical cancer
**Henley 2010[11]** Manuscript	USA	Retrospective cohort, National registry	2004–2006	National Program of Cancer Registries and SEER database	ICD-O-3: C53<br>Collaborative Stage classification	Localized, regional, distant	• Invasive cervical, breast, or colon/rectum cancers<br>• ≥20 years of age for cervical cancer	36 076
**Subramaniam 2010[41]** Congress abstract	Birmingham, Alabama, USA	Retrospective cohort, Single center institution	2002–2007	University-based gynecologic oncology program	NR	I, II, III, IV	• Invasive cervical cancer	430
**Garg 2011[10]** Manuscript	USA	Retrospective cohort, National registry	1988–2005	SEER database 17 registries used	NR<br>FIGO staging	IIA (IIA1, IIA2)	• Stage IIA cervical cancer<br>• Primary treatment with RH or RT	560
**McLean 2012[36]** Congress abstract	USA	Retrospective cohort, Healthcare database	1992–2007	SEER-Medicare database	NR	I, II, III, IV	• Any stage cervical cancer<br>• Aged 65–100 years	6718
**McLean 2012[35]** Congress abstract	USA	Case-control, Healthcare database	1992–2007	SEER-Medicare database	NR	I, II, III, IV	• Diagnosed with cervical cancer after age 70 (n=734)<br>• Matched non-cancer controls (n=2936)	734
**Skaznik-Wikiel 2012[22]** Manuscript	USA	Retrospective cohort, National registry	2000–2006	SEER database 17 registries used	NR<br>FIGO staging	I, II, III, IV	• Cervical cancer diagnosis	18 003

Study / publication type	Location	Study design & data source type	Time-frame	Data source	Classification criteria[a]	Stages of cervical cancer included	Population	Total patients with cervical cancer
**Ojha 2014[17]** Manuscript	USA	Longitudinal/cohort, National registry	1973–2010	SEER database Only 9 registries used: Atlanta, Connecticut, Detroit, Hawaii, Iowa, New Mexico, San Francisco-Oakland, Seattle-Puget Sound, Utah<br><br>SEER-PAYA cancer survivors' cohort	NR<br><br>SEER summary staging	1 – localized<br>2/3 – locally advanced<br>4 – metastatic	• PAYA: females diagnosed with any cancer before age 30 years, had survived ≥5 years post-diagnosis, and were later diagnosed with invasive cervical cancer (n=46)<br>• Females in the general population aged ≤56 years at primary cervical cancer diagnosis (n=26,956)	27 002
**Machida 2018[14]** Manuscript	USA	Retrospective cohort, National registry	1973–2013	SEER database	ICD-O-3 and WHO classifications (histology)<br><br>TNM: AJCC 7th ed. staging	I, II, III, IV	• Cervical cancer diagnosis	87 151
**Zahnd 2018[29]** Manuscript	USA	Retrospective cohort, National registry	2009–2013	North American Association of Central Cancer Registries	NR<br><br>SEER summary stage	Localized and distant	• All stageable cancer types combined<br>• HPV-associated cancers<br>• Tobacco-associated cancers	NR

Study / publication type	Location	Study design & data source type	Time-frame	Data source	Classification criteria[a]	Stages of cervical cancer included	Population	Total patients with cervical cancer
							• Individual cancers with screening recommendation from the United States Preventive Services Task Force and has current recommendations (colorectal, female breast, cervical, and lung)<br>• Cancers for which screening was recommended for most of the study period (prostate)<br>• Cancers with insufficient evidence for recommended screening but for which screening may be performed regularly in clinical practice (skin and oral)	
**Hou 2019[12]** Manuscript	USA	Retrospective cohort, National registry	1988–2011	SEER database	ICD-O-3: C53.0-53.9<br>FIGO staging	I, II, III, IV	• White and Asian-American patients with cervical cancer	58 780

Study / publication type	Location	Study design & data source type	Time-frame	Data source	Classification criteria[a]	Stages of cervical cancer included	Population	Total patients with cervical cancer
**Bruegl 2020[5]** Manuscript	Idaho, Oregon, Washington, USA	Retrospective cohort, Regional registry	1996–2016	Cancer Data Registry of Idaho Oregon State Cancer Registry Washington State Cancer Registry	ICD-O-3: C53.0-53.9<br>NR	Localized, regional, distant	• Non-Hispanic White and American Indian/Alaskan Native women diagnosed with a gynecological cancer	7222
**Tian 2020[24]** Manuscript	USA	Retrospective cohort, National registry	2010–2015	SEER database 18 registries used	NR<br>FIGO staging	IB2–IVA	• Cervical cancer stages IB2 to IVA<br>• Pathological biopsy confirmed SCC and AC<br>• No distant metastases<br>• Aged 20–69 years	4131
**Mahmud 2011[15]** Manuscript	Saskatchewan, Canada	Retrospective cohort, Regional registry	1987–2001	Provincial cancer registry-Saskatchewan	NR<br>FIGO staging	I, II, III, IV	• Cervical cancer diagnosis	714
**Popadiuk 2010[38]** Congress abstract	Newfoundland, Canada	Retrospective cohort, Regional registry	1992–2008	Newfoundland Cancer Registry	NR	IA, IB, IIB, IIIB, IVA	• Invasive cervical cancer<br>• Aged 19–29 years	37
**Carmo 2011[6]** Manuscript	Rio de Janeiro, Brazil	Retrospective cohort, Single center institution	1999–2004	Brazilian National Cancer Institute	NR<br>FIGO staging	I, II, III, IV	• Cervical cancer diagnosis	3341
**Rodrigues 2018[39]** Congress abstract	Brazil	Prospective, Multiple institutions	2016–2017	16 sites, representing 5 Brazilian regions	NR	I, II, III, IV	• Invasive cervical cancer<br>• Aged ≥18 years	631
**Possati-Resende 2018[18]** Manuscript	Barretos, Brazil	Retrospective cohort, Single center institution	2003–2015	Prevention Institute at Barretos Cancer Hospital	NR	I, II, III, IV	• Cervical cancer diagnosis	NR

Study / publication type	Location	Study design & data source type	Time-frame	Data source	Classification criteria[a]	Stages of cervical cancer included	Population	Total patients with cervical cancer
**Warner 2018[27]** Manuscript	Trinidad and Tobago	Retrospective cohort, National registry	1995–2009	Dr. Elizabeth Quamina Cancer Registry (aka National Cancer Registry of Trinidad and Tobago)	ICD-10: C53<br>NR	Localized, regional, distant	• Any cancer diagnosis	1812
**Nathani 2012[37]** Congress abstract	Bradford, UK	Retrospective cohort, Single center institution	2007–2011	Bradford Royal Infirmary database	NR	IA1, IA2, IB1, III	• Cervical cancer diagnosis<br>• Aged 19–30 years	19
**Garry 2018[31]** Congress abstract	Dublin, Ireland	Retrospective cohort, Single center institution	2006–2015	Electronic case report forms from a tertiary oncology center	NR<br>FIGO staging	IA, IB, II, III, IV	• Cervical cancer diagnosis<br>• Aged ≥60 years	119
**Lorin 2015[13]** Manuscript	Côte-d'Or, France	Retrospective cohort, Regional registry	1998–2010	Côte d'Or gynecological registry	NR<br>FIGO staging	I, II, III, IV	• Invasive cervical cancer	311
**Rottmann 2020[40]** Congress abstract + poster	Upper Bavaria, Germany	Retrospective cohort, Regional registry	2007–2016	Munich Cancer Registry	NR	IA1–IV, M1	• Cervical cancer diagnosis	2291
**Litvinova 2017[34]** Congress abstract	Minsk City, Belarus	Retrospective cohort, National registry	2012–2016	National Cancer Registry	NR	IIB, III, IVA	• Unresectable cervical cancer diagnosis<br>• Only young women discussed for proportions of disease by stage	324
**Samson 2016[19]** Manuscript	Bulgaria	Retrospective cohort, National registry	1993–2013	Bulgarian National Cancer Registry	ICD-O: C53.0, C53.1, C53.8, and C53.9	I, II, III, IV	• Cervical cancer diagnosis	21 737
**Ojamaa 2018[16]** Manuscript	Estonia	Retrospective cohort, National registry	1968–2014	Estonian Cancer Registry	ICD-O-3: C53.0; C53.1, C53.8, and C53.9	I, II, III, IV	• Invasive cervical cancer	3403

Monk BJ, *et al. Int J Gynecol Cancer* 2022; 32:1531–1539. doi: 10.1136/ijgc-2022-003801

**Study / publication type**	**Location**	**Study design & data source type**	**Time-frame**	**Data source**	**Classification criteria[a]**	**Stages of cervical cancer included**	**Population**	**Total patients with cervical cancer**
					TNM (AJCC 7th ed) for staging			
**Ulinskas 2013[25]** Manuscript	Lithuania	Retrospective cohort, National registry	1990–2004	Lithuanian cancer registry	ICD-10: C53.0, C53.1, C53.8, and C53.9<br><br>NR	I, II, III, IV	• Cervical cancer diagnosis	6680
**Kosgei 2018[33]** Congress abstract	Uasin Gishu, Kenya	Retrospective cohort, Regional registry	2010–2014	Eldoret Cancer Registry	NR	I, II, III, IV	• Cervical cancer diagnosis	265
**Bouchbika 2013[4]** Manuscript	Casablanca, Morocco	Retrospective cohort, Regional registry	2005–2007	Greater Casablanca Registry	ICD-O-3, converted to ICD-10: C53<br><br>NR	Localized, regional, distant	• Any cancer diagnosis	816
**Elmajjaoui 2016[9]** Manuscript	Morocco	Retrospective cohort, Multiple institutions	2006	National Institute of Oncology, Mohammed V Hospital, Rabat<br><br>Cheikh Khalifa Ibn Zaid Hospital, Université Mohammed VI des Sciences de la Santé, Casablanca	NR<br><br>FIGO staging	I, II, III, IV	• Invasive cervical cancer	646
**Somdyala 2020[23]** Manuscript	Eastern Cape Province, South Africa	Retrospective cohort, Regional registry	1998–2012	Eastern Cape Cancer Registry	ICD-O: C53.0–C53.9	I, II, III, IV	• Cervical cancer diagnosis	1315
**Sharkas 2017[20]** Manuscript	Jordan	Retrospective cohort, National registry	2000–2013	Jordan Cancer Registry	ICD-10: C53<br><br>TNM staging	Localized, regional, distant	• Cervical cancer diagnosis<br>• Only women who were "Jordanian"	591
**Kaidarova 2018[32]** Congress abstract	Kazakhstan	Retrospective cohort, National registry	2012	Kazakhstan Cancer Registry	NR	IA, IB, IIA, IIB, III	• Cervical cancer diagnosis	1641

Study / publication type	Location	Study design & data source type	Time-frame	Data source	Classification criteria[a]	Stages of cervical cancer included	Population	Total patients with cervical cancer
**Alkhalawi 2019[3]** Manuscript	Gulf countries	Retrospective cohort, Multinational registry	1998–2012	Gulf Centre for Cancer Control and Prevention Database	ICD-O-3: C53.0, C53.2, C53.8, C53.9<br><br>SEER summary staging	Localized, regional, distant	• Invasive cervical cancer	2332
**Agarwal 2012[2]** Manuscript	Delhi, India	Retrospective cohort, Single center institution	2000–2009	Guru Teg Bahadur Hospital	NR<br><br>FIGO staging	I, II, III, IV	• Any primary gynecologic cancer diagnosis	927
**Shruthi 2014[21]** Manuscript	Kolar, India	Retrospective cohort, Single center institution	NR<br>1-year period	Sri Devaraj Urs Medical College, Sri Devaraj Urs Academy of Higher Education and Research	NR<br><br>TNM staging	I, II, III, IV	• Cervical cancer diagnosis	199
**Wang 2015[26]** Manuscript	Beijing, China	Retrospective cohort, Regional registry	1993–2008	Statistics Database of Beijing Cancer Registry	ICD-O<br>FIGO staging	I, II, III, IV	• Cervical cancer diagnosis<br>• Beijing residents only	3641
**Cheung 2011[7]** Manuscript	Hong Kong, China	Retrospective cohort, Regional registry	1997–2006	Hong Kong Cancer Registry	NR<br><br>FIGO and TNM staging	I, II, III, IV	• Cervical cancer diagnosis	4407
**Yagi 2019[28]** Manuscript	Osaka Prefecture, Japan	Retrospective cohort, Regional registry	1976–2012	Osaka Cancer Registry	C53, C54, C55 (C55 later sorted to C53 or C54 using a multiple imputation estimation)<br><br>TNM staging	Localized (T1N0M0), regional lymph nodes (N1), adjacent organs (T2, 3, 4), distant (M1)	• Cervical cancer diagnosis	25 826
**Seol 2014[30]** Congress abstract	Korea	Retrospective cohort, National registry	1999–2010 (total population)<br><br>1999-2004 (with stage information)	Korea Central Cancer Registry	NR<br><br>FIGO staging	IA1-IVB	• Cervical cancer diagnosis	49 503 (total population)<br><br>19 282 (with stage information)

BMJ Publishing Group Limited (BMJ) disclaims all liability and responsibility arising from any reliance placed on this supplemental material which has been supplied by the author(s)

**Study / publication type**	**Location**	**Study design & data source type**	**Time-frame**	**Data source**	**Classification criteria[a]**	**Stages of cervical cancer included**	**Population**	**Total patients with cervical cancer**
				Gynecologic Oncology Committee of Korean Society of Obstetrics and Gynecology				
**Chiang 2016[8]** Manuscript	Taiwan	Retrospective cohort, National registry	2002–2012	Taiwan Cancer Registry	*ICD-O-3*: C53<br>TNM staging	I, II, III, IV	• Any invasive cancer<br>• Age ≥15 years	8238

[a]Two types of classifications were found in the included studies. Disease coding classification criteria was used to identify patients with cervical cancer in large registries and databases and included different versions of the ICD or ICD-O criteria. The specific codes used to identify cervical cancer patients are also summarized where available. The second classification types found in the included studies were used to determine the stage of disease, and included FIGO, TNM, SEER summary, and Collaborative Stage criteria.

AC, adenocarcinoma; AJCC, American Joint Committee on Cancer; FIGO, International Federation of Gynecology and Obstetrics; HPV, human papillomavirus; *ICD-10*, *International Classification of Diseases, 10th edition; ICD-O, International Classification of Diseases*, *Oncology*; NR, not reported; PAYA, pediatric and young adult cancers; RH, radical hysterectomy; RT, radiotherapy; SCC, squamous cell carcinoma; SEER, Surveillance, Epidemiology, and End Results; TNM, tumor, node, metastasis; USA, United States of America; WHO, World Health Organization.

Supplementary Table 6. Studies reporting incidence by stage of cervical cancer.

Reference	Region	Location / data collection period	N	Incidence of cervical cancer by stage	Incidence of locally advanced cervical cancer
Bruegl 2020[5][a]	North America	USA Idaho, Oregon, Washington 1996–2016	7222	*Age-standardized rate per 100 000 population*<br><br>*American Indian/Alaskan Natives*<br>Localized, 4.3<br>Regional, 3.6<br>Distant, 1.8<br>Unknown, 0.9<br><br>*Non-Hispanic White*<br>Localized, 3.7<br>Regional, 2.0<br>Distant, 0.7<br>Unknown, 0.5	*Age-standardized rate per 100 000 population*<br><br>*American Indian/Alaskan Natives*<br>Regional, 3.6<br><br>*Non-Hispanic White*<br>Regional, 2.0
Henley 2010[11]	North America	USA 2004–2006	36,076	*Age-standardized rate per 100 000 population*<br><br>Localized, 5.3<br>Regional, 4.0<br>Distant, 1.2<br>Unknown, 0.9	*Age-standardized rate per 100 000 population*<br><br>Regional, 4.0
Zahnd 2018[29][b]	North America	USA 2009–2013	Not reported	*Age-standardized rate per 100 000 population*<br><br>*Rural*<br>Localized, 3.7<br>Distant, 1.1<br><br>*Urban*<br>Localized, 3.4<br>Distant, 1.0	Not calculable
McClean 2012[36]	North America	USA 1992–2007	6718	*Women aged 65–100 years, Age-adjusted incidence rate*<br><br>Stage I, decreased by 2.4% per year	*Women aged 65–100 years, Age-adjusted incidence rate*<br><br>Stage III, increased by 2.0% per year

				Stage III, increased by 2.0% per year	
Litvinova 2017[34]	Europe	Belarus Minsk City 2012–2016	324	*Incidence per 100 000 female population*<br><br>IIB, decreased from 3.8 to 1.9<br>III, decreased from 3.2 to 2.3<br>IVA, increased from 0.4 to 0.7	*Incidence per 100 000 female population*<br><br>IIB, decreased from 3.8 to 1.9<br>III, decreased from 3.2 to 2.3<br>IVA, increased from 0.4 to 0.7

[a]The Bruegl 2020 study only included patients who were *American Indian/Alaskan Natives* or non-Hispanic White.
[b]In the Zahnd 2018 study, only the incidence of localized and distant cervical cancer was compared in urban and rural areas; neither of these stages was considered locally advanced disease according to our method of estimation (ie, only “regional” disease is considered).

USA, United States of America.

Section and Topic	Item #	PRISMA Checklist item	Location where item is reported
**TITLE**			
Title	1	Identify the report as a systematic review.	Page 1
**ABSTRACT**			
Abstract	2	See the PRISMA 2020 for Abstracts checklist.	Page 3
**INTRODUCTION**			
Rationale	3	Describe the rationale for the review in the context of existing knowledge.	Page 5
Objectives	4	Provide an explicit statement of the objective(s) or question(s) the review addresses.	Page 5
**METHODS**			
Eligibility criteria	5	Specify the inclusion and exclusion criteria for the review and how studies were grouped for the syntheses.	Pages 5-6, Supplementary Appendix pages 4-5
Information sources	6	Specify all databases, registers, websites, organisations, reference lists and other sources searched or consulted to identify studies. Specify the date when each source was last searched or consulted.	Page 5, Supplementary Appendix pages 1-3
Search strategy	7	Present the full search strategies for all databases, registers and websites, including any filters and limits used.	Supplementary Appendix pages 1-3
Selection process	8	Specify the methods used to decide whether a study met the inclusion criteria of the review, including how many reviewers screened each record and each report retrieved, whether they worked independently, and if applicable, details of automation tools used in the process.	Page 6
Data collection process	9	Specify the methods used to collect data from reports, including how many reviewers collected data from each report, whether they worked independently, any processes for obtaining or confirming data from study investigators, and if applicable, details of automation tools used in the process.	Page 6
Data items	10a	List and define all outcomes for which data were sought. Specify whether all results that were compatible with each outcome domain in each study were sought (e.g. for all measures, time points, analyses), and if not, the methods used to decide which results to collect.	Supplementary Appendix page 5
	10b	List and define all other variables for which data were sought (e.g. participant and intervention characteristics, funding sources). Describe any assumptions made about any missing or unclear information.	Supplementary Appendix pages 5
Study risk of bias assessment	11	Specify the methods used to assess risk of bias in the included studies, including details of the tool(s) used, how many reviewers assessed each study and whether they worked independently, and if applicable, details of automation tools used in the process.	N/A
Effect measures	12	Specify for each outcome the effect measure(s) (e.g. risk ratio, mean difference) used in the synthesis or presentation of results.	N/A
Synthesis methods	13a	Describe the processes used to decide which studies were eligible for each synthesis (e.g. tabulating the study intervention characteristics and comparing against the planned groups for each synthesis (item #5)).	Supplementary Appendix page 5

Section and Topic	Item #	PRISMA Checklist item	Location where item is reported
	13b	Describe any methods required to prepare the data for presentation or synthesis, such as handling of missing summary statistics, or data conversions.	Page 6-7, Supplementary Appendix page 5-6
	13c	Describe any methods used to tabulate or visually display results of individual studies and syntheses.	Page 6-7, Supplementary Appendix page 5-6
	13d	Describe any methods used to synthesize results and provide a rationale for the choice(s). If meta-analysis was performed, describe the model(s), method(s) to identify the presence and extent of statistical heterogeneity, and software package(s) used.	Page 6-7, Supplementary Appendix page 5-6
	13e	Describe any methods used to explore possible causes of heterogeneity among study results (e.g. subgroup analysis, meta-regression).	Supplementary Appendix page 8
	13f	Describe any sensitivity analyses conducted to assess robustness of the synthesized results.	N/A
Reporting bias assessment	14	Describe any methods used to assess risk of bias due to missing results in a synthesis (arising from reporting biases).	N/A
Certainty assessment	15	Describe any methods used to assess certainty (or confidence) in the body of evidence for an outcome.	N/A
**RESULTS**			
Study selection	16a	Describe the results of the search and selection process, from the number of records identified in the search to the number of studies included in the review, ideally using a flow diagram.	Page 7 and Figure 1
	16b	Cite studies that might appear to meet the inclusion criteria, but which were excluded, and explain why they were excluded.	N/A
Study characteristics	17	Cite each included study and present its characteristics.	Pages 7-8, Supplementary figure 1, Supplementary Tables 3, 4, 5
Risk of bias in studies	18	Present assessments of risk of bias for each included study.	N/A
Results of individual studies	19	For all outcomes, present, for each study: (a) summary statistics for each group (where appropriate) and (b) an effect estimate and its precision (e.g. confidence/credible interval), ideally using structured tables or plots.	Pages 8-11, Figure 2, Tables 1-3, Supplementary Figure 2

Monk BJ, *et al. Int J Gynecol Cancer* 2022; 32:1531–1539. doi: 10.1136/ijgc-2022-003801

Section and Topic	Item #	PRISMA Checklist item	Location where item is reported
Results of syntheses	20a	For each synthesis, briefly summarise the characteristics and risk of bias among contributing studies.	Pages 8-11, Figure 2, Tables 1-3
	20b	Present results of all statistical syntheses conducted. If meta-analysis was done, present for each the summary estimate and its precision (e.g. confidence/credible interval) and measures of statistical heterogeneity. If comparing groups, describe the direction of the effect.	Pages 8-11, Figure 2, Tables 1-3, Supplementary Figure 2
	20c	Present results of all investigations of possible causes of heterogeneity among study results.	Page 9, Supplementary Figure 2
	20d	Present results of all sensitivity analyses conducted to assess the robustness of the synthesized results.	N/A
Reporting biases	21	Present assessments of risk of bias due to missing results (arising from reporting biases) for each synthesis assessed.	N/A
Certainty of evidence	22	Present assessments of certainty (or confidence) in the body of evidence for each outcome assessed.	N/A
**DISCUSSION**			
Discussion	23a	Provide a general interpretation of the results in the context of other evidence.	Pages 11-12
	23b	Discuss any limitations of the evidence included in the review.	Page 12-13
	23c	Discuss any limitations of the review processes used.	Page 12-13
	23d	Discuss implications of the results for practice, policy, and future research.	Page 13
**OTHER INFORMATION**			
Registration and protocol	24a	Provide registration information for the review, including register name and registration number, or state that the review was not registered.	Page 6
	24b	Indicate where the review protocol can be accessed, or state that a protocol was not prepared.	Page 6, Supplementary Appendix page 1
	24c	Describe and explain any amendments to information provided at registration or in the protocol.	N/A
Support	25	Describe sources of financial or non-financial support for the review, and the role of the funders or sponsors in the review.	Page 15
Competing interests	26	Declare any competing interests of review authors.	Page 15
Availability of data, code and other materials	27	Report which of the following are publicly available and where they can be found: template data collection forms; data extracted from included studies; data used for all analyses; analytic code; any other materials used in the review.	N/A

From: Page MJ, McKenzie JE, Bossuyt PM, Boutron I, Hoffmann TC, Mulrow CD, et al. The PRISMA 2020 statement: an updated guideline for reporting systematic reviews. BMJ 2021;372:n71. doi: 10.1136/bmj.n71

For more information, visit: http://www.prisma-statement.org/

REFERENCES

1 Ruhl JL, Callaghan C, Hurlbut A*, et al.*, editors. Summary Stage 2018: Codes and Coding Instructions. Bethesda, MD: National Cancer Institute; 2020.

2 Agarwal S, Malhotra KP, Sinha S*, et al.* Profile of gynecologic malignancies reported at a tertiary care center in India over the past decade: comparative evaluation with international data. *Indian J Cancer* 2012;49:298-302. doi: 10.4103/0019-509X.104494

3 Alkhalawi E, Al-Madouj A, Al-Zahrani A. Cervical cancer incidence and trends among Nationals of the Gulf Cooperation Council States, 1998-2012. *Gulf J Oncolog* 2019;1:7-13. doi:

4 Bouchbika Z, Haddad H, Benchakroun N*, et al.* Cancer incidence in Morocco: report from Casablanca registry 2005-2007. *Pan Afr Med J* 2013;16:31. doi: 10.11604/pamj.2013.16.31.2791

5 Bruegl AS, Joshi S, Batman S*, et al.* Gynecologic cancer incidence and mortality among American Indian/Alaska Native women in the Pacific Northwest, 1996-2016. *Gynecol Oncol* 2020;157:686-92. doi: 10.1016/j.ygyno.2020.03.033

6 Carmo CC, Luiz RR. Survival of a cohort of women with cervical cancer diagnosed in a Brazilian cancer center. *Rev Saude Publica* 2011;45:661-7. doi: 10.1590/s0034-89102011005000029

7 Cheung FY, Mang OW, Law SC. A population-based analysis of incidence, mortality, and stage-specific survival of cervical cancer patients in Hong Kong: 1997-2006. *Hong Kong Med J* 2011;17:89-95. doi:

8 Chiang CJ, Lo WC, Yang YW*, et al.* Incidence and survival of adult cancer patients in Taiwan, 2002-2012. *J Formos Med Assoc* 2016;115:1076-88. doi: 10.1016/j.jfma.2015.10.011

9 Elmajjaoui S, Ismaili N, El Kacemi H*, et al.* Epidemiology and outcome of cervical cancer in national institute of Morocco. *BMC Womens Health* 2016;16:62. doi: 10.1186/s12905-016-0342-2

10 Garg G, Shah JP, Toy EP*, et al.* Stage IIA1 versus stage IIA2 cervical cancer: does the new staging criteria predict survival? *Int J Gynecol Cancer* 2011;21:711-6. doi: 10.1097/IGC.0b013e3182138648

11 Henley SJ, King JB, German RR*, et al.* Surveillance of screening-detected cancers (colon and rectum, breast, and cervix) - United States, 2004-2006. *MMWR Surveill Summ* 2010;59:1-25. doi:

12 Hou Y, Guo S, Lyu J*, et al.* Prognostic factors in Asian and white American patients with cervical cancer, considering competing risks. *Curr Oncol* 2019;26:e277-e85. doi: 10.3747/co.26.4473

13 Lorin L, Bertaut A, Hudry D*, et al.* About invasive cervical cancer: a French population based study between 1998 and 2010. *Eur J Obstet Gynecol Reprod Biol* 2015;191:1-6. doi: 10.1016/j.ejogrb.2015.04.007

14 Machida H, Blake EA, Eckhardt SE*, et al.* Trends in single women with malignancy of the uterine cervix in United States. *J Gynecol Oncol* 2018;29:e24. doi: 10.3802/jgo.2018.29.e24

15 Mahmud A, Brydon B, Tonita J*, et al.* A population-based study of cervix cancer: incidence, management and outcome in the Canadian province of Saskatchewan. *Clin Oncol (R Coll Radiol)* 2011;23:691-5. doi: 10.1016/j.clon.2011.05.002

16 Ojamaa K, Innos K, Baburin A*, et al.* Trends in cervical cancer incidence and survival in Estonia from 1995 to 2014. *BMC Cancer* 2018;18:1075. doi: 10.1186/s12885-018-5006-1

17 Ojha RP, Jackson BE, Tota JE*, et al.* Younger age distribution of cervical cancer incidence among survivors of pediatric and young adult cancers. *Gynecol Oncol* 2014;134:309-13. doi: 10.1016/j.ygyno.2014.05.011

18 Possati-Resende JC, Vazquez FL, Biot ST*, et al.* Organized cervical cancer screening program in Barretos, Brazil: experience in 18 municipalities of Sao Paulo State. *Acta Cytol* 2018;62:19-27. doi: 10.1159/000480446

19 Samson KK, Haynatzki G, Soliman AS*, et al.* Temporal changes in the cervical cancer burden in Bulgaria: Implications for eastern european countries going through transition. *Cancer Epidemiol* 2016;44:154-60. doi: 10.1016/j.canep.2016.08.014

20 Sharkas G, Arqoub K, Khader Y*, et al.* Trends in the incidence of cervical cancer in Jordan, 2000-2013. *J Oncol* 2017;2017:6827384. doi: 10.1155/2017/6827384

21 Shruthi PS, Kalyani R, Kai LJ*, et al.* Clinicopathological correlation of cervical carcinoma: a tertiary hospital based study. *Asian Pac J Cancer Prev* 2014;15:1671-4. doi: 10.7314/apjcp.2014.15.4.1671

22 Skaznik-Wikiel ME, Sukumvanich P, Austin RM*, et al.* Heavy cervical cancer burden in elderly women: how can we improve the situation? *Acta Cytol* 2012;56:388-93. doi: 10.1159/000338555

23 Somdyala NIM, Bradshaw D, Dhansay MA*, et al.* Increasing cervical cancer incidence in rural Eastern Cape Province of South Africa from 1998 to 2012: a population-based cancer registry study. *JCO Glob Oncol* 2020;6:1-8. doi: 10.1200/JGO.19.00198

24 Tian T, Gong X, Gao X*, et al.* Comparison of survival outcomes of locally advanced cervical cancer by histopathological types in the surveillance, epidemiology, and end results (SEER) database: a propensity score matching study. *Infect Agent Cancer* 2020;15:33. doi: 10.1186/s13027-020-00299-3

25 Ulinskas K, Aleknaviciene B, Smailyte G. Demographic differences in cervical cancer survival in Lithuania. *Open Medicine* 2013;8:16-21. doi: doi:10.2478/s11536-012-0051-7

26 Wang T, Wu MH, Wu YM*, et al.* A population-based study of invasive cervical cancer patients in Beijing: 1993-2008. *Chin Med J (Engl)* 2015;128:3298-304. doi: 10.4103/0366-6999.171420

27 Warner WA, Lee TY, Badal K*, et al.* Cancer incidence and mortality rates and trends in Trinidad and Tobago. *BMC Cancer* 2018;18:712. doi: 10.1186/s12885-018-4625-x

28 Yagi A, Ueda Y, Kakuda M*, et al.* Epidemiologic and clinical analysis of cervical cancer using data from the population-based Osaka Cancer Registry. *Cancer Res* 2019;79:1252-9. doi: 10.1158/0008-5472.CAN-18-3109

29 Zahnd WE, Fogleman AJ, Jenkins WD. Rural-urban disparities in stage of diagnosis among cancers with preventive opportunities. *Am J Prev Med* 2018;54:688-98. doi: 10.1016/j.amepre.2018.01.021

30 Seol H-J, Ki K-D, Lee J-M. Epidemiologic characteristics of cervical cancer in Korean women. *J Gynecol Oncol* 2014;25:70-4. doi: http://dx.doi.org/10.3802/jgo.2014.25.1.70

31 Garry N, Corbett G, Thompson C*, et al.* Examining the effects that a woman's age, histological subtype and FIGO stage has on the treatment strategies and survival outcomes in cervical cancer. *Int J Gynecol Cancer* 2018;28:275. doi: 10.1136/00009577-201809002-00001

32 Kaidarova D, Chingissova Z, Adilbay D*, et al.* Five-year overall survival in patients with cervical cancer in Kazakhstan. *Int J Gynecol Cancer* 2018;28:302. doi: 10.1136/00009577-201809002-00001

33 Kosgei A, Chesumbai G, Buziba N*, et al.* Cervical cancer incidence and trends in Uasin Gishu County, Kenya (2010 to 2014). *JCO Glob Oncol* 2018;4:192s-s. doi: 10.1200/jgo.18.79501

34 Litvinova T, Matylevich O, Kosenko I*, et al.* Distinctive features of unresectable uterine cervix cancer in Belarus. *Int J Gynecol Cancer* 2017;27:825. doi: 10.1097/01.IGC.0000527296.86225.87

35 McLean K, Van Cleve W, Eckert L*, et al.* Associations between Papanicolaou testing and cervical cancer in elderly women. *Gynecologic Oncol* 2012;127:S11. doi: 10.1016/j.ygyno.2012.07.031

36 McLean K, Van Cleve W, Eckert L*, et al.* Cervical cancer incidence and screening patterns in elderly women. *Int J Gynecol Cancer* 2012;22:E676. doi:

37 Nathani F, Whelan C, Fayre A*, et al.* Cervical cancer in young women - Bradford Royal Infirmary. *BJOG* 2012;119:158-67. doi: https://doi.org/10.1111/j.1471-0528.2012.03379.x

38 Popadiuk C, Rose J. Does delaying the onset of pap smear screening in Newfoundland (NL) impact on cervical cancer rates? *J Clin Oncol* 2010;28:e12002-e. doi: 10.1200/jco.2010.28.15_suppl.e12002

39 Rodrigues MF, de Melo AC, Calabrich A*, et al.* Social disparities and patients' attitudes are associated with lower rates of cervical cancer screening in Brazil: results of EVITA study (LACOG 0215). *J Clin Oncol* 2018;36:e17510. doi: 10.1200/JCO.2018.36.15_suppl.e17510

40 Rottmann M, Schubert-Fritschle G, Engel J. Prognostic factors and outcomes of cervical cancer patients (2007-2016): a population-based analysis. *Oncol Res Treat* 2020;43:90-1. doi: 10.1159/000506491

41 Subramaniam A, Fauci JM, Schneider KE*, et al.* Invasive cervical cancer and screening: what are the rates of unscreened and underscreened women in the modern era? *J Low Genit Tract Dis* 2010;14:250. doi: 10.1097/LGT.0b013e3181eb2087
